# Compact, Polyvalent Mannose Quantum Dots as Sensitive, Ratiometric FRET Probes for Multivalent Protein–Ligand Interactions

**DOI:** 10.1002/anie.201600593

**Published:** 2016-03-16

**Authors:** Yuan Guo, Chadamas Sakonsinsiri, Inga Nehlmeier, Martin A. Fascione, Haiyan Zhang, Weili Wang, Stefan Pöhlmann, W. Bruce Turnbull, Dejian Zhou

**Affiliations:** ^1^ School of Chemistry and Astbury Centre for Structural Molecular BiologyUniversity of LeedsLeedsLS2 9JTUK; ^2^ Department of ChemistryUniversity of YorkHeslingtonYorkYO10 5DDUK; ^3^ Infection Biology UnitGerman Primate CenterKellnerweg 437077GöttingenGermany

**Keywords:** carbohydrates, FRET, proteins, quantum dots, viral inhibition

## Abstract

A highly efficient cap‐exchange approach for preparing compact, dense polyvalent mannose‐capped quantum dots (QDs) has been developed. The resulting QDs have been successfully used to probe multivalent interactions of HIV/Ebola receptors DC‐SIGN and DC‐SIGNR (collectively termed as DC‐SIGN/R) using a sensitive, ratiometric Förster resonance energy transfer (FRET) assay. The QD probes specifically bind DC‐SIGN, but not its closely related receptor DC‐SIGNR, which is further confirmed by its specific blocking of DC‐SIGN engagement with the Ebola virus glycoprotein. Tuning the QD surface mannose valency reveals that DC‐SIGN binds more efficiently to densely packed mannosides. A FRET‐based thermodynamic study reveals that the binding is enthalpy‐driven. This work establishes QD FRET as a rapid, sensitive technique for probing structure and thermodynamics of multivalent protein–ligand interactions.

Over the past 15 years, quantum dot (QD) Förster resonance energy transfer (FRET) technology has emerged as a powerful tool to address a broad range of biomedical questions because it combines the spectroscopic ruling ability of FRET and the stable, bright fluorescence of QDs.^1^ It has been widely used for bio‐/enzymatic/environmental/intracellular sensing, bio‐diagnostics, cell monitoring and tracking.^1^, ^2^ Despite great progress, QD FRET has not been applied to probe multivalent protein–ligand interactions which are widespread and crucial for many important biological events such as viral infection, immune response, cell signaling, and its regulation.^3^ This limitation is primarily due to a lack of an effective approach to prepare compact (hydrodynamic diameter, *D_h_*<10 nm), biocompatible and dense polyvalent QDs which are essential for multivalent binding and sensitive FRET readout.1b Compact, biocompatible QDs have been prepared by cap‐exchange using dihydrolipoic acid (DHLA) based ligands for sensing and imaging applications.^4^ The requirement of using a large excess of ligand (e.g. ligand:QD molar ratio of 10^4^–10^5^:1) of current protocols,^4^ however, makes it impractical to initiate direct QD cap‐exchange using expensive custom ligands. Thus functional groups are mostly conjugated to cap‐exchanged QDs using various coupling and bioconjugation approaches. It has been difficult to achieve high polyvalency (>150) on compact, sub‐10 nm QDs. Herein we have solved this problem by performing cap‐exchange in a homogeneous solution using functional ligands appending a deprotonated DHLA moiety. Our approach greatly improved the cap‐exchange efficiency, allowing for production of compact, dense polyvalent mannose‐capped QDs using 20–200 fold less ligand than literature protocols. We demonstrate that such compact, polyvalent mannose‐capped QDs can provide quantitative binding affinity and thermodynamic parameters for multivalent protein–glycan interactions underpinning HIV/Ebola viral infections through a sensitive, ratiometric FRET readout strategy.

Here the dendritic cell receptor, DC‐SIGN (one of the most important cell pathogen receptors)^5^ and an endothelial cell receptor, DC‐SIGNR, were employed as model multimeric proteins. These proteins recognize multiple mannose‐containing glycans on the human immunodeficiency virus (HIV) and Ebola virus (EBOV) surface glycoproteins via their clustered carbohydrate‐recognition‐domains (CRDs, Figure 1 E).^5^ The resulting high affinity, multivalent binding can enhance viral infectivity. It is known that synthetic multivalent glycoconjugates can inhibit such interactions.^6^ Despite sharing 77 % amino acid identity and an overall tetrameric structure, DC‐SIGN/R have shown to possess notable differences in glycan binding affinity, specificity and viral transmission efficiency. For example, DC‐SIGN recognizes and transmits some HIV strains more effectively than DC‐SIGNR,^7^ whereas only DC‐SIGNR promotes West Nile Virus (WNV) infection with high efficiency.^8^ Given individual CRD–mannose binding motifs are identical in DC‐SIGN/R5b and the binding affinities are very weak (*K*_D_≈mm),^9^ such differences must stem from their different multivalent binding properties which are still poorly understood. We reasoned that a polyvalent mannose–QD conjugate would be useful for probing the CRD arrangements here because it combines features of weak individual CRD–mannose binding affinity and nanoscale spherical geometry. As a result, only the protein with CRDs facing the same direction can bind to the QD multivalently, leading to high affinity.

**Figure 1:**
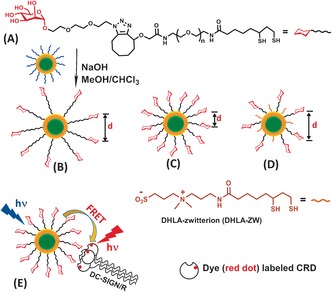
A) Chemical structure of the dihydrolipoic acid–poly(ethylene glycol)–mannose (DHLA‐PEG_n_‐Man) ligands and the schematic of our ligand exchange approach using mannose‐capped quantum dots. B,C,D) Tuning interglycan spacing by a PEG linker length of DHLA‐PEG_*n*_‐Man [*n*≈13 (B) or 3 (C)] and diluting with a DHLA‐zwitterion spacer ligand (D). E) Cartoon of probing multivalent interactions between extracellular segment of DC‐SIGN/R and QD‐PEG_*n*_‐Man by FRET.

First, two multifunctional mannose‐containing ligands were designed and synthesized (see the Supporting Information for details). Each ligand comprises a DHLA moiety for strong chelative binding to QD surface Zn^2+^ ions; a poly(ethylene glycol), PEG, linker for resisting non‐specific adsorption and imposing high stability and biocompatibility;^4^ and a mannose residue for specific protein binding (abbreviated as DHLA‐PEG_n_‐Man hereafter, where *n*=3 or about 13 stands for uniform or mixed length linker containing 3 or an average of 13 PEG units, respectively, Figure 1 A).

The DHLA‐PEG_*n*_‐Man ligands were subsequently employed to perform cap‐exchange with hydrophobic CdSe/ZnS QDs (4.2 nm diameter, *λ*_EM_ ca. 560 nm; see Figure S1A in the Supporting Information) to make QD‐PEG_*n*_‐Man probes. Cap‐exchange was performed in homogenous solution (e.g. 1:1 v/v CHCl_3_/MeOH) using deprotonated DHLA to facilitate the ligand exchange process and enhance their QD binding affinity because thiolates bind much more strongly to Zn^2+^ ions than thiols (see Section A4 in the Supporting Information).4a Under such conditions, stable, biocompatible QD‐PEG_*n*_‐Mans were readily prepared at a ligand:QD molar ratio of 500:1, a substantial 20–200 fold lower than literature protocols. Importantly, this improvement made it practical to directly initiate cap‐exchange with hydrophobic QDs using precious functional mannose ligands. It also enabled us to achieve unprecedented levels of high glycan polyvalency (ca. 330±70 and 170±30 for QD‐EG_3_‐Man and QD‐PEG_13_‐Man) on compact sub‐10 nm QDs (Figure S1). This would be very difficult for other current literature methods. Furthermore, this method also facilitated tuning the density and spacing of glycans at the QD surface by dilution with a DHLA‐zwitterion ligand (Figure 1 D). These advantageous properties made the QDs powerful FRET probes for investigating multivalent protein–glycan interactions for the first time. Interestingly, the average inter‐Man distance in the QD‐EG_3_‐Man was estimated as about 0.98 nm (see Section A43 in the Supporting Information), matching well to the inter‐glycosylation spacing of about 1 nm found on the HIV surface glycoprotein, gp120.^10^

To probe the multivalent binding by FRET, DC‐SIGN was labeled with Atto‐594 dye (Section A51/52) on a site‐specifically introduced cysteine residue. The dye labeling did not affect its specific binding to a Sepharose‐mannose column. The Atto‐594‐QD FRET pair had a respectable Förster radius (*R_0_*=4.7/5.0 nm for QD‐EG_3_‐Man/QD‐PEG_13_‐Man, respectively; see Figure S2). Binding of the labeled DC‐SIGN to the QDs yielded significantly reduced QD fluorescence at 554 nm together with concurrently enhanced Atto‐594 FRET signal at 626 nm (Figure 2 A,B), which was fully consistent with a QD‐sensitized Atto‐594 FRET mechanism. Stronger FRET signals and more severely quenched QD fluorescence were observed for DC‐SIGN binding to QD‐EG_3_‐Man over QD‐PEG_13_‐Man, indicating more efficient FRET in the former pair. Both bindings displayed excellent fits (*R* ^2^>0.99) by the single QD donor FRET with N identical acceptors model,^2^ yielding QD–dye distances (*r*) of about 6.8/9.8 nm for QD‐EG_3_‐Man/QD‐PEG_13_‐Man, respectively (Figure S3). These *r* values roughly matched the sum of QD core radius plus respective fully extended ligand length (ca. 6.5 and 10.0 nm; Figure S1).

**Figure 2:**
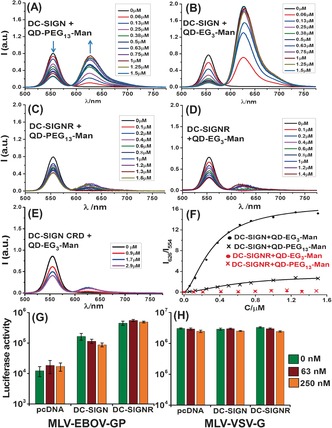
A–E) Acceptor direct excitation background‐corrected fluorescence spectra of QD‐PEG_*n*_‐Man (*λ*_EM_=554 nm, final C_QD_=40 nm) after binding to Atto‐594‐labeled proteins: A) DC‐SIGN+QD‐PEG_13_‐Man; B) DC‐SIGN+QD‐EG_3_‐Man; C) DC‐SIGNR+QD‐PEG_13_‐Man; D) DC‐SIGNR+QD‐EG_3_‐Man; E) DC‐SIGN CRD monomer+QD‐EG_3_‐Man. F) Relationship between the apparent FRET ratio (I_626_/I_554_) and protein concentration fitted to the Hill equation. G,H) Luciferase activities of cell lysates of DC‐SIGN/R expressing 293T cells after exposure to an Ebola virus glycoprotein‐bearing, luciferase‐encoding murine leukemia virus (MLV‐EBOV‐GP) vector in the presence of indicated amounts of QD‐EG_3_‐Man in Dulbecco's modified eagle medium supplemented with 10 % fetal bovine serum. A MLV vector bearing the vesicular stomatitis virus glycoprotein (MLV‐VSV‐G) was used as negative control. Cells transfected with empty plasmid (pcDNA) were used as additional negative controls. In panel (G), the reduction of EBOV‐GP‐dependent transduction by 250 nm QD‐EG_3_‐Man was statistically significant from the 0 nm QD control (*p*=0.024).

The observed FRET signal was completely diminished in the absence of Ca^2+^ (Figure S4), suggesting the signal was indeed the result of Ca^2+^‐dependent DC‐SIGN‐mannose binding.^11^ Moreover, the FRET signal was effectively inhibited by free mannose in a dose‐dependent manner, while galactose was much less effective at inhibiting this binding (Figure S5). This result agrees well with DC‐SIGN's binding specificity for mannose over galactose.^11^ A higher mannose concentration (*K*_I_) was required to inhibit DC‐SIGN binding to QD‐EG_3_‐Man than to QD‐PEG_13_‐Man (1.4 vs. 0.80 mm, see Table 1), which was consistent with the former binding being tighter (apparent *K*_D_ 0.32 vs. 0.6 μm).

**Table 1:** Biophysical and thermodynamic parameters of the DC‐SIGN‐QD interactions.

QD probe	Apparent *K*_D_ [mm]	*K*_I_ [mm]^[a]^	Δ*H* [kJ mol^−1^]	Δ*S* [J K^−1^ mol^−1^]
QD‐PEG_13_‐Man	0.6±0.1	0.8±0.1	−44±1	−40±2
QD‐EG_3_‐Man	0.32±0.07	1.4±0.1	−56±6	−55±18

[a] Inhibition constant of DC‐SIGN‐QD binding by free mannose.

Surprisingly, binding of DC‐SIGNR (also labeled with Atto‐594, Section A51) to the QDs yielded only very weak FRET signals (Figure 2 C and D) which were barely stronger than that of the monomeric CRD (Figure 2 E) or non‐specific interaction between DC‐SIGN/R and a DHLA‐ZW‐capped control QD (Figure S6), suggesting minimal binding. Despite some degree of QD quenching being observed for the DC‐SIGNR and control samples, the specific QD‐DC‐SIGN binding was clearly distinguished from these controls through analysis of the FRET ratio (I_626_/I_554_) which is linearly correlated to the amounts of QD‐bound proteins (Section A55). The apparent FRET ratio for DC‐SIGN followed typical binding curves (Figure 2 F and SI, Table S1). However, the signals for DC‐SIGNR remained low and comparable to non‐specific interaction throughout the concentrations tested (Figure S6G). In fact, the maximum I_626_/I_554_ value for DC‐SIGN binding to QD‐PEG_13_‐Man/ QD‐EG_3_‐Man was 12/60 times greater than that of the equivalent DC‐SIGNR binding, demonstrating a remarkable binding specificity of the QDs for DC‐SIGN over DC‐SIGNR, two closely related tetrameric receptors having almost identical protein sequence. To our knowledge, this level of DC‐SIGN/R discrimination (ca. 60‐fold) is unprecedented for polyvalent ligands built upon such simple carbohydrates. This work thus demonstrates the role of polyvalency in determining multivalent binding selectivity, and opens up a new method for understanding glycobiology where multivalent effects are absolutely essential to biological activity.

The QD‐DC‐SIGN binding specificity was further verified by a cell based assay. Here, a murine leukemia virus (MLV) vector was used to deliver the luciferase gene to human embryonic kidney cells (293T) previously transfected to express DC‐SIGN/R. The MLV vector bearing Ebola virus glycoprotein (EBOV‐GP) can bind to cell surface DC‐SIGN/R to augment cell entry and gene transduction.5a,5d DC‐SIGN/R expression in cells markedly increased the gene transduction efficiency. While QD‐EG_3_‐Man treatment significantly reduced the gene transduction of DC‐SIGN‐positive cells in a dose‐dependent manner (Figure 2 G), presumably via binding to cell surface DC‐SIGN, which blocked the binding and entry of the EBOV‐GP‐bearing vector. In contrast, gene transduction of cells expressing DC‐SIGNR was unaffected by QD‐EG_3_‐Man. This inhibiting specificity matched perfectly with the QD's much higher affinity to DC‐SIGN over DC‐SIGNR. Finally, QD‐EG_3_‐Man did not modulate significantly the gene transduction of control cells (pcDNA) nor the transduction driven by a control vector bearing vesicular stomatitis virus glycoprotein (VSV‐G) which cannot use DC‐SIGN/R for cell entry (Figure 2 H).5a,5d These results confirmed that the specific QD‐DC‐SIGN binding was responsible for the observed inhibition.

High mannose density appears to favor binding to DC‐SIGN over DC‐SIGNR. Consistent with this, the high glycan density on HIV (each gp120 contains 25 glycosylation sites)^10^ also favors DC‐SIGN binding/transfection over WNV whose glycoprotein contains just 1 glycosylation site.^12^ Diluting the QD surface DHLA‐EG_3_‐Man density with DHLA‐ZW strongly affected its DC‐SIGN binding. The I_626_/I_554_ ratios all increased linearly with the increasing protein concentration, except for the 100 % QD‐EG_3_‐Man at high concentration due to surface binding saturation (Figure 3 A). Since the I_626_/I_554_ ratio is linearly correlated to the amounts of QD‐bound proteins (Section A55), the slopes of the binding curves thus represents the binding efficiency (or fraction of added proteins that have bound to the QD). Note, not all added DC‐SIGNs may bind to the QD due to natural binding/dissociation equilibrium. The QD‐DC‐SIGN binding efficiency was decreased rapidly with mannose ligand dilution (Figure 3 B), revealing a strong preference of DC‐SIGN for binding to multivalent ligands with a high mannose density.

**Figure 3:**
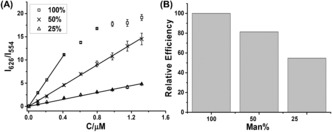
A) A plot of apparent FRET ratio (I_626_/I_554_) as a function of DC‐SIGN concentration for QD‐EG_3_‐Man capped with different percentage of DHLA‐EG_3_‐Man ligand: square (100 %); cross (50 %) and triangle (25 %). B) Normalized DC‐SIGN binding efficiency per mannose for QD‐EG_3_‐Man at different surface mannose density.

The FRET ratio for DC‐SIGN binding to both QDs was found to decrease with increasing temperature (Figure S7). Assuming that the maximum binding (ca. I_626_/I_554_) was independent of temperature, then apparent *K*_D_s at each temperature were obtained (Table S2). The binding thermodynamic parameters were obtained from an Arrhenius data analysis (Table 1). The QD‐DC‐SIGN binding was found to be enthalpy driven, with QD‐EG_3_‐Man giving greater negative enthalpy and entropy changes. Individual CRD–mannose binding was also found to be enthalpy‐driven from an isothermal titration calorimetry study.^9^ Thus the same binding mechanism may be involved in the multivalent QD‐DC‐SIGN binding.

The apparent *K*_D_s for DC‐SIGN‐QD binding were all in the high nm range (Table 1), >5000‐fold tighter than individual mannose–CRD binding (*K*_D_=3.5 mm),^9^ indicating that multivalent binding greatly enhanced the binding affinity. Because the mannose moieties are covalently coupled to a solid, non‐deformable and spherical QD core, only receptors having CRDs that face in the same direction are able to bind multivalently to the QD. The minimal DC‐SIGNR‐QD binding revealed here implies an inability to form effective multivalent binding. Based on their distinct QD‐binding properties, we propose that the CRDs are facing upwardly along the coiled‐coil axes in DC‐SIGN (hence readily accessible to multivalent binding to the QD), but sideways in DC‐SIGNR (hence unavailable to bind the QD multivalently, Figure 4). Such structural models agree well with those proposed from small‐angle X‐ray scattering studies.^13^ The different CRD arrangement and accessibility in DC‐SIGN/R may account for their distinct viral binding/transmission properties. It also correlates well with the biological roles: the high accessibility of DC‐SIGN should enable rapid antigen capture to trigger the immune response as required for an antigen‐presenting dendritic cell surface endocytic receptor.^11^, ^14^ Whereas the endothelial cell surface adhesion receptor DC‐SIGNR^11^ may only recognize specific, spatial and orientation‐matched multivalent glycans.

**Figure 4:**
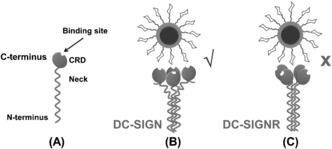
Schematics of the different DC‐SIGN/R‐QD‐Man multi‐valent binding. A) Schematic structure of a DC‐SIGN/R subunit. B) The uprightly facing DC‐SIGN CRDs readily bind to multiple mannoses on the QD, resulting in strong multivalent binding. C) The sideway pointing CRDs in DC‐SIGNR cannot bind to multiple sugars on the QD simultaneously, leading to weak/minimal binding.

In conclusion, an efficient ligand exchange approach for making compact, biocompatible QDs densely capped with specific glycans has been developed, enabling QD FRET to be employed to probe multivalent receptor–glycan interactions for the first time. Compared to other biophysical techniques, QD FRET has the advantages of high sensitivity, rapid, separation‐free detection in solution, and ratiometric readout signal, rendering detection highly robust and reliable. It can provide quantitative binding thermodynamics and reveal insights of binding site arrangement and accessibility in multimeric proteins. In particular, we reveal that binding sites arrangements in DC‐SIGN and DC‐SIGNR are functionally distinct and only DC‐SIGN binds efficiently to small, spherical multivalent glycan ligands. We further demonstrate that the polyvalent QD specifically inhibits DC‐SIGN‐, but not DC‐SIGNR‐mediated pseudo‐Ebola virus entry of target cells in serum media. This work establishes a potential new strategy for targeting DC‐SIGN‐ from DC‐SIGNR‐mediated viral infection.

## Supporting information

As a service to our authors and readers, this journal provides supporting information supplied by the authors. Such materials are peer reviewed and may be re‐organized for online delivery, but are not copy‐edited or typeset. Technical support issues arising from supporting information (other than missing files) should be addressed to the authors.

Supplementary
