# Supplementary material for: Compact, Polyvalent Mannose Quantum Dots as Sensitive, Ratiometric FRET Probes for Multivalent Protein–Ligand Interactions
Source: Angew Chem Int Ed Engl. 2016 Mar 16;55(15):4738–42. doi: 10.1002/anie.201600593 (PMC4979658; doi:10.1002/anie.201600593)
Supplement: Supplementary file 1 — Supplementary [file ANIE-55-4738-s001.pdf]

Angewandte Chemie
Eine Zeitschrift der Gesellschaft Deutscher Chemiker

Supporting Information

Compact, Polyvalent Mannose Quantum Dots as Sensitive, Ratiometric FRET Probes for Multivalent Protein–Ligand Interactions

Yuan Guo, Chadamas Sakonsinsiri, Inga Nehlmeier, Martin A. Fascione, Haiyan Zhang, Weili Wang, Stefan Pöhlmann, W. Bruce Turnbull, and Dejian Zhou**

anie_201600593_sm_miscellaneous_information.pdf

Contents

- Material, instrument and methods page 2
- Synthesis and characterization of lipoic acid-zwitterion (LA-ZW) and lipoic acid polyethylene glycol-mannose (LA-PEG-Man) page 4
- Preparation and characterization of QD-PEG_n-Man page 13
- Protein Production, Labeling and FRET Analysis page 14
- Pseudo-viral inhibition assay page 18
- Supporting Tables page 19
- Supporting Figures page 20
- References page 26

A1: Materials

CdSe/ZnS core/shell QD with λ_{EM} of 560 nm was purchased commercially from PlasmaChem GmbH (Berlin, Germany). The QD was supplied as dry powders capped with mixed hydrophobic ligands of trioctyl-phosphione oxide (TOPO), hexadecylamine and oleic acid. Polyethylene glycol (PEG) with an average molecular weight of 600 (containing an average of ~13 PEG units, denoted as PEG_{13}); 11-Azido-3,6,9-trioxaundecan-1-amine (> 90% monomer purity), N,N-dimethyl-1,3-propanediamine (>99%), 1,3-propane-sultone (> 99%), triphenylphosphine (>98.5%), dicyclohexylcarbodiimide (DCC, >99%), dimethylamino-pyridine (DMAP, >99%), lipoic acid (LA,> 99%), tris(2-carboxyethyl)phosphine hydrochloride (TCEP.HCl, >98%), triethylamine (>99%), chloroform (> 99.8%), magnesium sulfate (>99%), methanol (>99.9%), potassium hydroxide, ethyl acetate (>99.0%), methylene chloride (>98%) sodium bicarbonate (>99.5%) and other chemicals were purchased from Sigma-Aldrich (Dorset, UK) and used as received without further purification unless stated otherwise. Solvents were obtained from Fisher Scientific (Loughborough, UK) and used as received. Ultra-pure water (resistance >18.2 MΩ.cm) purified by an ELGA Purelab classic UVF system, was used for all experiments and making buffers.

A2) Instrument and Methods[1-3]

All moisture-sensitive reactions were performed under nitrogen atmosphere using oven-dried glassware. Dry solvents were obtained through an innovative technology solvent drying system. Evaporations were performed under reduced pressure on a rotary evaporator. The synthesis was monitored by TLC on silica gel 60 F254 plates on aluminum and stained by iodine. Flash column chromatography was performed on silica gel 60 A (Merck grade 9385). All ^{1}H and ^{13}C NMR spectra are recorded on Brucker DPX300 (500 MHz for ^{1}H, 125 MHz for ^{13}C) in $CDCl_3$ except for azide-modified mannoses which were recorded on a 300 MHz machine (75 MHz for ^{13}C NMR). All chemical shifts were reported in parts per million (ppm). The coupling constants were given in Hz. Assignment of ^{1}H NMR spectra was achieved using 2D methods (COSY) when necessary. The following abbreviations are used in ^{1}H NMR analysis: s = singlet, brs = broad singlet, d = doublet, t = triplet, q = quartet, m = multiplet, dd = double doublet, dt = double triplet, td = triple doublet, ddd = double double doublet.

High resolution mass spectra (HR-MS) were obtained on a Bruker Daltonics MicroTOF mass spectrometer. Infra-red spectra were recorded on a PerkinElmer FT-IR spectrometer. Melting points were obtained on a Griffin melting point apparatus. Optical rotations were measured at the sodium D-line with a Schmidt + Haensch Polartronic H532 polarimeter. $[\alpha]_D$ values were given in units of 10^{-1} deg cm^2 g^{-1}. UV-vis absorption spectra were recorded on a Varian Cary 50 bio UV-Visible Spectrophotometer over 200-800 nm using 1 mL quartz cuvette with an optical path of 1 cm or on a Nanodrop 2000 spectrophotometer (Thermo scientific) over the range of 200-800 nm using 1 drop of the solution with an optical path length of 1 mm. All centrifugations were carried out on a Thermo Scientific Heraeus Fresco 21 microcentrifuge using 1.5 mL microcentrifuge tubes at room temperature (unless stated otherwise). The QD purification was performed by Amicon ultra-centrifugal filter tubes with a cut-off MW of 30,000. Dynamic light scattering (DLS) was measured using Zetasizer Nano (Malvern) at a QD-Man concentration of 40 nM as decribed previously.[2]

All fluorescence spectra were measured on a Spex Fluoro Max-3 Spectrofluorometer using a 0.70 mL quartz cuvette under a fixed excitation wavelength of 450 nm. This wavelength corresponds to the absorption mimimum of the Atto-594 dye to minimize the background resulting from direct excitation of the acceptor.[1] An excitation and emission bandwidths of 5 nm and a scan rate of 120 nm/min over 480-800 nm range were used. All measurements were done in the binding buffer (20 mM HEPES, 100 mM NaCl, 10 mM $CaCl_2$ pH 7.8) containing 10 μg/mL of a His_6-Cys peptide which we have previously found it increased the stability of the QD.[1] The QDs were mixed with the calculated amounts of labelled proteins thoroughly in binding buffer at room temperature for 20 min (all final QD concentration, C_{QD} = 40 nM) before fluorescence measurement. For sugar competition assay, mannose (Man) or galactose (Gal) stock was first made in the binding buffer. Different amounts of Man or Gal were mixed with the labeled DC-SIGN (final $C_{protein}$ = 0.5 μM) first before adding to the QD-Man conjugates (final C_{QD} = 40 nM). The mixture was incubated at room temperature for 20 min before measurement.

A3) Ligand Synthesis and Chacterisation

A31: Synthesis of lipoic acid-zwitterion ligand (LA-ZW)[4]

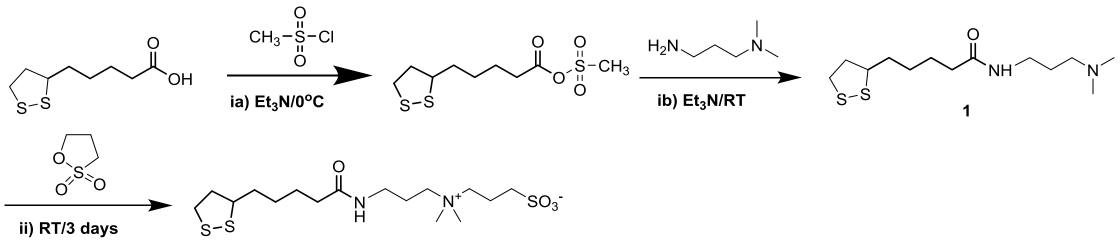

LA-ZW

2

Scheme S1. **The synthetic route to LA-ZW**. Reaction conditions are: **ia**) Et_3N, CH_2Cl_2, MsCl; **ib**) N,N-dimethyl-1,3-propanediamine, Et_3N/RT; **ii**) $CHCl_3$, 1,3-propanesultone, RT/3 days

Step i) Synthesis of LA-N, N-dimethyl-1, 3-propanediamine.

Lipoic acid (LA, 3.0 g, ~15 mmol), triethylamine (1.47 g, ~15 mmol) and 30 mL CH_2Cl_2 were mixed in three-necked bottom flask (250 mL) and then cooled to 0 °C in an ice-bath under N_2 under stirring for 30 mins. Methanesulfonyl chloride (1.67 g, ~15 mmol) was the added dropwisely through a syringe (1 mL) and the resulting solution was allowed to slowly warm up to room temperature (RT) and stirred for 5 h. After that, N,N-dimethyl-1,3-propanediamine (1.24 g, ~12 mmol) and triethylamine (0.61 g, ~6 mmol) dissolved in 20 mL CH_2Cl_2 was slowly added. The resulting solution was stirred at overnight under N_2 at RT. The reaction mixture was then transferred to a separation funnel and washed with water (30 mL × 2) and then saturated Na_2CO_3 solution (100 mL). The organic layer was dried over Na_2SO_4 and filtered. After evaporation of the solvent, the desired compound (**1**) was obtained as a yellow oil 1.48 g, yield 34.7%. ^{1}H NMR (500 MHz, $CDCl_3$): δ (ppm): 5.45 (s, 1H), 3.50-3.59 (m,1H), 3.30 (m, 2H), 3.05-3.20 (m, 2H), 2.38-2.49 (m, 1H), 2.38 (t, 2H, J = 6 Hz), 2.20 (s, 6H), 2.17 (t, 2H, J = 6 Hz), 1.83-1.94 (m, 1H), 1.57-1.72 (m, 6H), 1.38-1.52 (m, 2H).

Step ii) Synthesis of LA-zwitterion (LA-ZW) ligand.

Compound **1** (1.48 g, ~5.2 mmol) was dissolved in 20 mL dry THF and purged with N_2 for 30 mins. Then 1,3-propanesultone (1.0 g, ~8 mmol) dissolved in 4 mL dry THF was added through a syringe. The resulting mixture was stirred at RT for 3 days. A turbidity was instantly noticed as 1,3-propanesultone was added, indicating the formation of LA-zwitterion (**2**) due to its low solubility in THF. After stirring for 3 days, the solvent was evaporated to yield the crude product as a pale yellow solid. The crude product was washed with chloroform (20 mL × 3) and further purified by HPLC to give the pure TA-zwitterion (**2**) in 23% yield. ^{1}H NMR (300 MHz, D_2O): δ (ppm) 3.60-3.70 (m,1H), 3.40-3.50 (m, 2H), 3.28-3.35 (m, 2H), 3.20-3.28 (m, 2H), 3.10-3.20 (m, 2H), 3.10 (s, 6H), 2.90 (t, 2H), 2.40-2.50 (m, 1H), 2.20 (t, 2H), 2.15 (m, 2H), 1.93-2.0 (m, 2H), 1.70 (m, 1H), 1.50-1.60 (m, 4H), 1.35-1.40 (m, 2H).

A32: Synthesis of LA-PEG$_{13}$-Man and LA-EG$_3$-Man ligands

(a): Synthesis of azido-mannose derivatives[5,6]

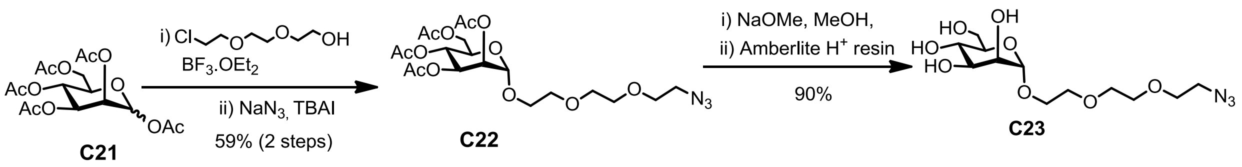

Scheme S2: Synthetic route to the azido-EG_2-mannose derivative.

Step 1: 1-Azido-3,6-dioxaoct-8-yl 2,3,4,6-tetra-*O*-acetyl α-D-mannopyranoside (C22)[5]

Freshly distilled boron trifluoride diethyl etherate (23.70 mL, 192.2 mmol) was added dropwise to a solution of mannose pentaacetate **C21** (25.0 g, 64 mmol), and 2-[2-(2-chloroethoxy)ethoxy]ethanol (18.62 mL, 128.1 mmol) in anhydrous dichloroethane (250 mL) at 0 °C. The reaction mixture was heated to 50 °C and stirred for 22 h. The reaction mixture was washed with saturated $NaHCO_3$ (250 mL × 2), brine (250 mL × 2), dried over Mg_2SO_4 and then concentrated to give brown crude oil. The compound was partially purified by flash column chromatography (silica; 1:1 hexane/EtOAc). The partially separated product (2.0 g, 4.0 mmol) was then dissolved in anhydrous DMF (100 mL) into which sodium azide (1.3 mg, 20 mmol) and tetrabutyl-ammonium iodide (1.48 g, 4 mmol) were added and then stirred at 87 °C for 17 h. The solvent was removed and the residue was purified by flash column chromatography (silica; 1:1 petroleum ether/EtOAc) to give compound **C22** (1.02 g, 59%, 2 steps) as colourless oil; R_f 0.16 (1:1 petroleum ether/EtOAc); $[\alpha]_D^{22}$ 41.01 (c, 2.2, $CHCl_3$) (lit.[3] $[\alpha]_D^{20}$ 61.5 (c, 0.22, $CHCl_3$). δ_H (500 MHz, $CDCl_3$); 5.33 (dd, 1H, $J_{2,3}$ 10.0 Hz, $J_{3,4}$ 3.5 Hz, H-3), 5.28-5.22 (m, 2H, H-2, H-4), 4.84 (d, 1H, $J_{1,2}$ 1.8 Hz, H-1), 4.26 (dd, 1H, $J_{6,6'}$ 12.2 Hz, $J_{5,6}$ 5.0 Hz, H-6), 4.07-4.01 (m, 2H, H-5, H-6'), 3.82-3.74 (m, 1H, ManOC$\underline{H}_2$), 3.69-3.59 (m, 9H, ManOC$\underline{H}_2$, 4 × C$\underline{H}_2$O), 3.40-3.34 (m, 2H, C$\underline{H}_2$-N), 2.12 (s, 3H, C(O)CH_3), 2.07 (s, 3H, C(O)CH_3), 2.01 (s, 3H, C(O)CH_3), 1.96 (s, 3H, C(O)CH_3); δ_C (75 MHz, $CDCl_3$); 170.6, 170.0, 169.9, 169.7 (4 × C=O), 97.7 (C-1), 70.8, 70.7, 70.1, 70.1 (4 × $\underline{C}H_2$O), 69.6 (C-2), 68.4 (ManO$\underline{C}H_2$), 67.4 (C-5), 69.1, 66.2 (C-3, C-4), 62.4 (C-6), 50.7 ($\underline{C}H_2$-N), 20.9, 20.8, 20.8, 20.7 (4 × C(O)$\underline{C}H_3$); IR (λ_{max}/cm^{-1}): 1741 (C=O), 2106 (N_3); HR-MS: Found $[M+Na]^+$ 528.1805, $C_{20}H_{31}N_3NaO_{12}$ requires 528.1800. The ^1H and ^{13}C NMR spectra were shown in the page 6.

Step 2: 1-Azido-3,6-dioxaoct-8-yl α-D-mannopyranoside (C23)[6]

Sodium methoxide (103 mg, 2.0 mmol) was added to a solution of compound **C22** (1.20 g, 2.4 mmol) in anhydrous MeOH (50 mL). After stirring for 3 h at RT, the reaction mixture was diluted with MeOH and neutralised with Amberlite® IRC 86 H^+ resin, filtered, re-dissolved in hot MeOH, cooled down, filtered through Celite® and then concentrated to afford compound **C23** (0.72 g, 90%) as a colourless oil. R_f 0.10 (9:1 DCM–MeOH); $[\alpha]_D^{22}$ 54.24 (c, 0.25, MeOH) (lit. value[4] $[\alpha]_D^{25}$ 38.7 (c, 1.0, MeOH); δ_H (500 MHz, D_2O); 4.91 (d, 1H, J 1.6 Hz, H-1), 3.99 (dd, 1H, $J_{2,3}$ 3.5 Hz, $J_{1,2}$ 1.6 Hz, H-2), 3.92-3.88 (m, 2H, ManOC$\underline{H}_2$), 3.86-3.83 (m, 1H, H-3), 3.80-3.66 (m, 12H, H-4, , H-5, H-6, H-6', 4 × OC$\underline{H}_2$), 3.52 (t, 2H, J 5.1 Hz, C$\underline{H}_2$-N); δ_C (75 MHz, D_2O); 100.0 ($J_{C,H}$ 168.9 Hz, α, C-1), 72.7 (C-4), 70.5 (C-3), 70.0 (C-2), 69.6, 69.5, 69.5, 69.3 (4 × $\underline{C}H_2$O), 66.7 (C-5), 66.4 (ManO-$\underline{C}H_2$), 60.9 (C-6), 50.2 ($\underline{C}H_2$-N); **IR** (λ_{max}/cm^{-1}): 3373 (OH), 2106 (N_3); **HRMS**: Found $[M+Na]^+$ 360.1377, $C_{12}H_{23}N_3NaO_8$ requires 360.1381. The corresponding ^1H and ^{13}C NMR spectra were shown in the page 7.

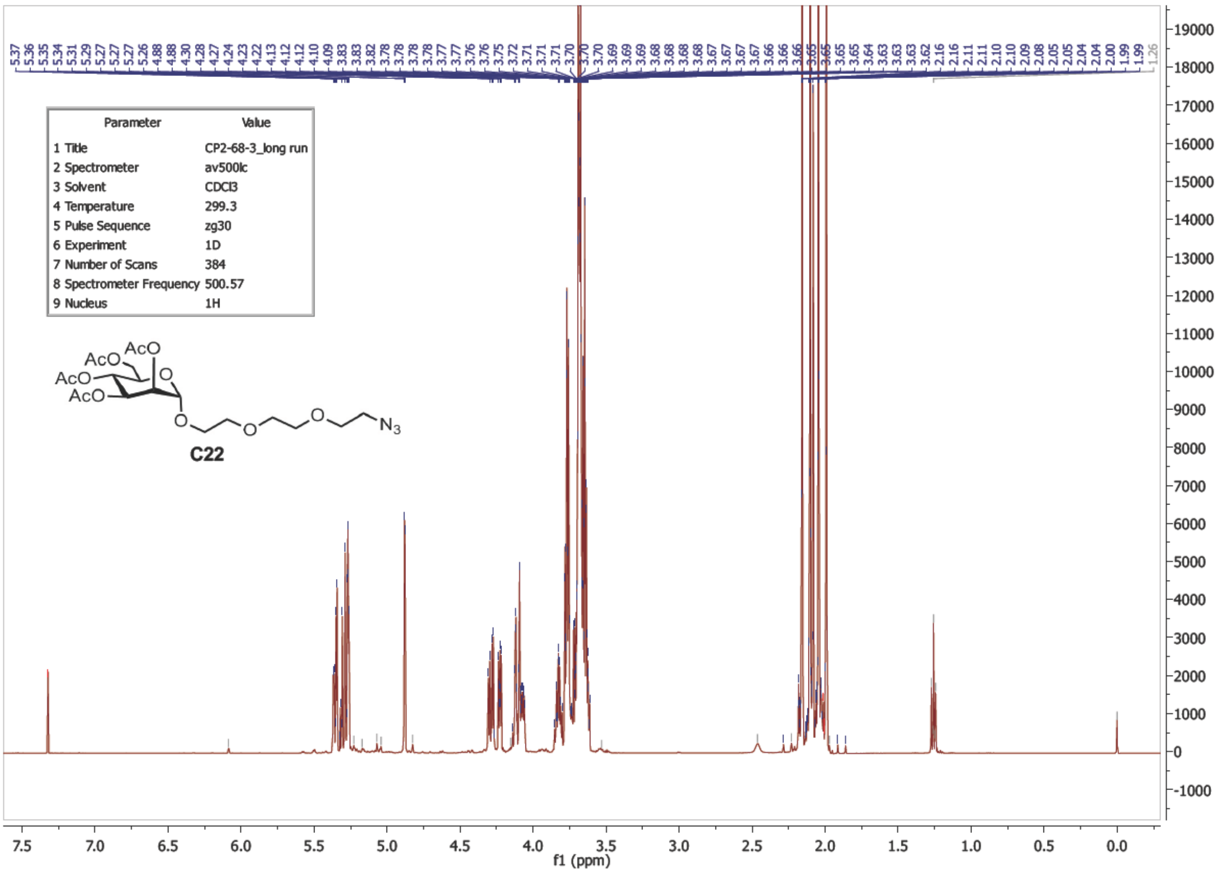
Parameter Value
1 Title CP2-68-3_long run
2 Spectrometer av500lc
3 Solvent CDCl3
4 Temperature 299.3
5 Pulse Sequence zg30
6 Experiment 1D
7 Number of Scans 384
8 Spectrometer Frequency 500.57
9 Nucleus 1H
C22
f1 (ppm)

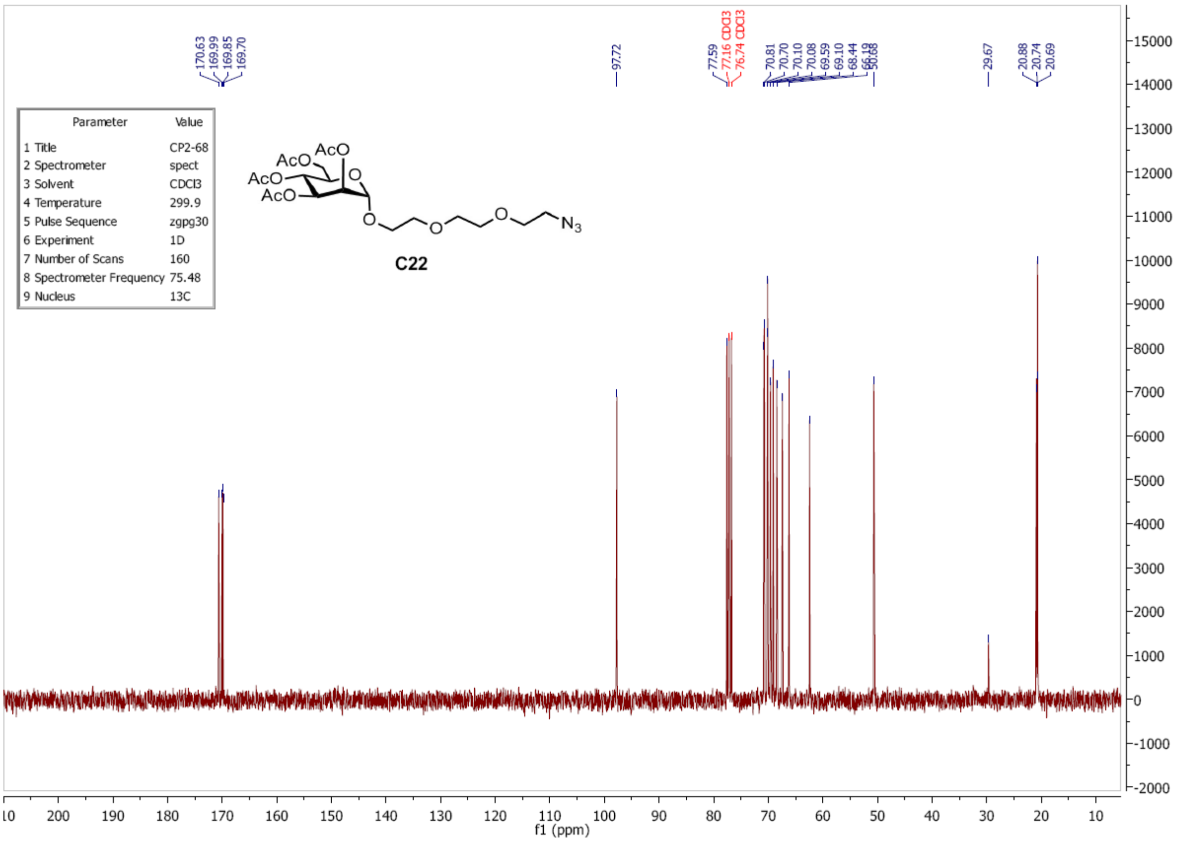
Parameter Value
1 Title CP2-68
2 Spectrometer spect
3 Solvent CDCl3
4 Temperature 299.9
5 Pulse Sequence zgpg30
6 Experiment 1D
7 Number of Scans 160
8 Spectrometer Frequency 75.48
9 Nucleus 13C
C22
f1 (ppm)

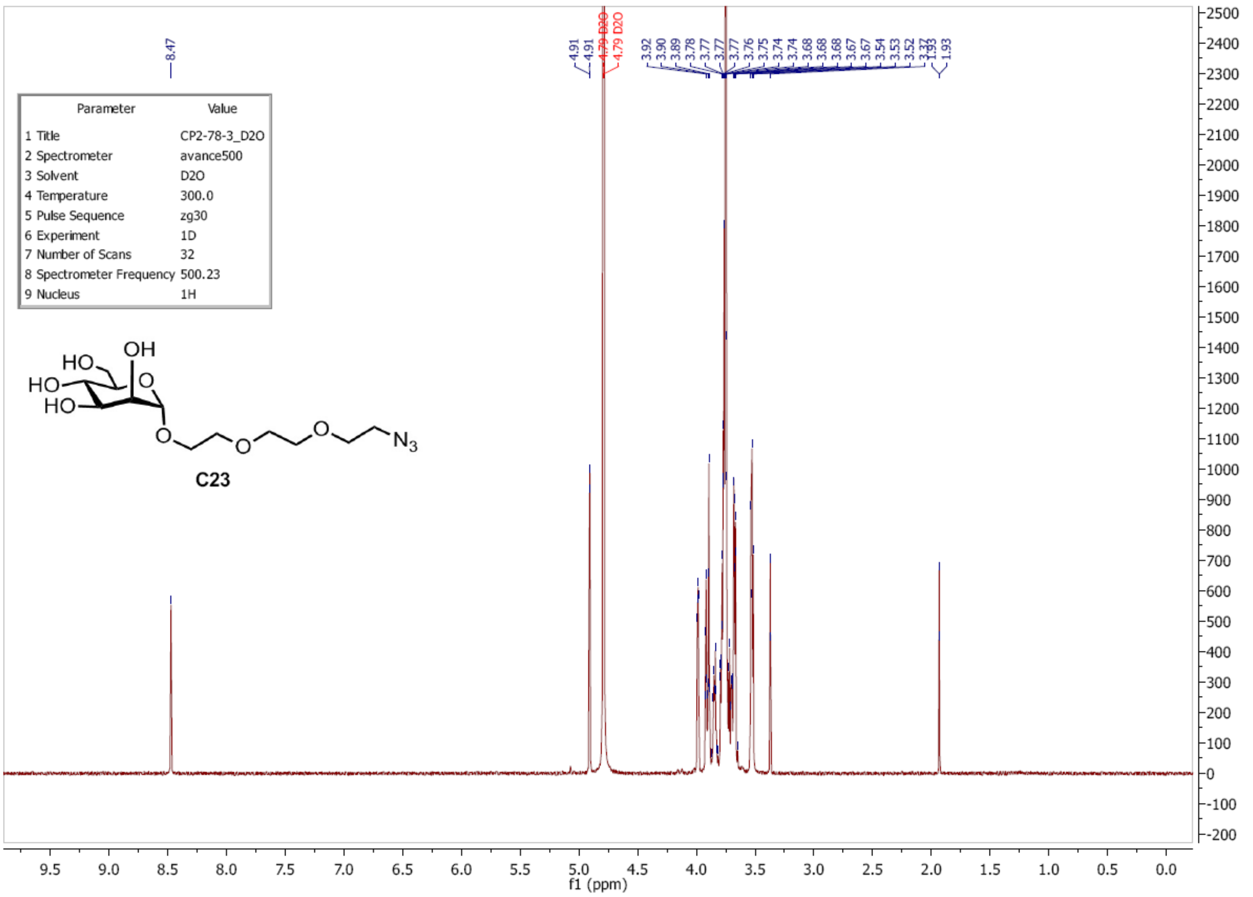
8.47
4.91
4.91
4.79 D2O
4.79 D2O
3.92
3.90
3.89
3.78
3.77
3.77
3.77
3.76
3.75
3.74
3.74
3.68
3.68
3.67
3.54
3.53
3.52
3.37
1.93
1.93
Parameter Value
1 Title CP2-78-3_D2O
2 Spectrometer avance500
3 Solvent D2O
4 Temperature 300.0
5 Pulse Sequence zg30
6 Experiment 1D
7 Number of Scans 32
8 Spectrometer Frequency 500.23
9 Nucleus 1H
HO
OH
HO
O
HO
O
O
O
N3
C23
f1 (ppm)

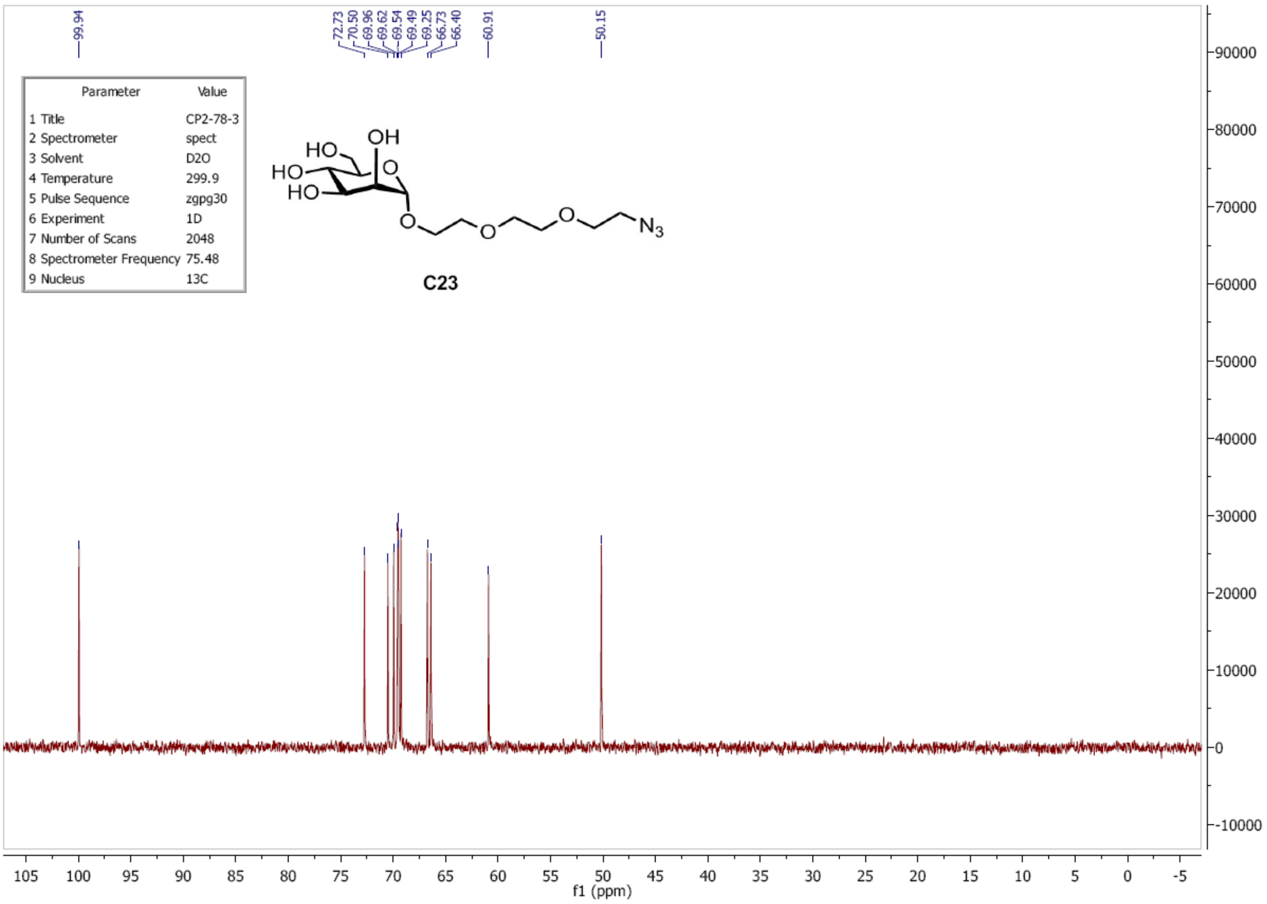
99.94
72.73
70.50
69.96
69.62
69.54
69.49
69.25
66.73
66.40
60.91
50.15
Parameter Value
1 Title CP2-78-3
2 Spectrometer spect
3 Solvent D2O
4 Temperature 299.9
5 Pulse Sequence zgpg30
6 Experiment 1D
7 Number of Scans 2048
8 Spectrometer Frequency 75.48
9 Nucleus 13C
HO
OH
HO
O
HO
O
O
O
N3
C23
f1 (ppm)

(b) Synthesis of LA-PEG13-Man and LA-EG3-Man[1,7]

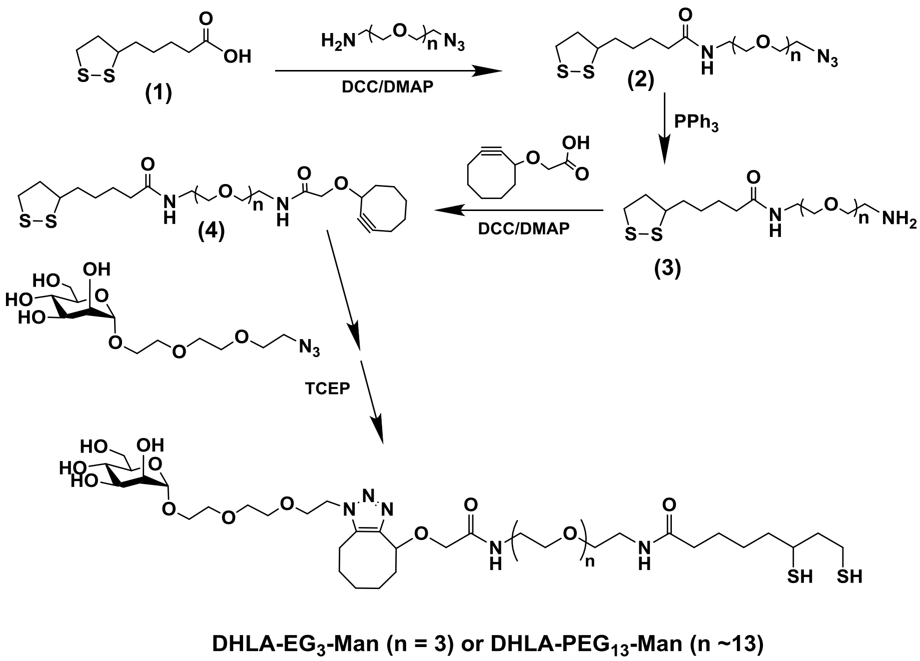

Scheme S3. The synthetic route to DHLA-PEG13-Man and DHLA-EG3-Man

Step 1: Synthesis of LA-PEGn-N3 (where n = ~13 or 3)

N_3-PEG13-NH_2, which is made of mixed length PEGs with an average length of 13 EG units, was synthesized and purified as described in our previous publication.[1] N_3-PEG13-NH_2 (6.5 g, 9.8 mmol contain), 4-N,N-dimethylaminopyridine (DMAP, 0.241 g, 1.98 mmol), N,N'-dicyclohexylcarbodiimide (DCC, 2.07 g, 10.1 mmol) and CH_2Cl_2 (60 mL) were added to a 250 mL 2 necked round-bottomed flask under a N_2 atmosphere. The mixture was cooled to 0 °C in an ice bath and lipoic acid (2.02 g, 9.8 mmol) in 32 mL of CH_2Cl_2 was then added dropwise over 30 min under constant stirring. After the addition was complete, the reaction mixture was allowed to warmed up to RT and stirred overnight. The mixture was then filtered off through Celite® and the solid was rinsed with $CHCl_3$. The filtrate was combined and evaporated to dryness, and then added with 100 mL H_2O. The resulting solution was washed with diethyl ether (100 mL × 2). The aqueous phase was saturated with $NaHCO_3$ and extracted with $CHCl_3$ (100 mL × 3). The combined organic layers were dried over $MgSO_4$. After evaporation of solvent, the residue was purified by silica gel column chromatography using 20:1 (vol/vol) $CHCl_3$: MeOH as eluting solvent. Each fraction was checked by TLC and the pure product fractions were combined ($CHCl_3$: MeOH =10:1 (vol/vol), $R_{f\ (LA\text{-}PEG13\text{-}N3)}$ = 0.52, R_f (TA) = 0.6, $R_{f\ (N3\text{–}PEG13\text{–}NH2)}$ = 0.04) and evaporated to dryness to give LA-PEG13-N_3 (**2**) as a yellow oil (2.30 g, yield: 28%). ^1H NMR (400 MHz, $CDCl_3$): δ 6.42 (br s, 1H), 3.4–3.5 (m), 3.36 (t, 2H, *J*=5.0 Hz), 3.26 (t, 2H, *J*=5.0 Hz), 3.0 (t, 2H, *J* = 5.2 Hz), 2.8–2.9 (m, 2H), 2.2 (m, 1H), 2.0 (t, 2H, *J* = 7.4 Hz), 1.71 (m, 1H), 1.5 (m, 4H), 1.27 (m, 2H). IR (neat): 3331; 3072; 2868; 2105; 1651; 1543; 1454; 1107 cm^{-1}.

A slightly modified procedure was used to synthesize LA-EG3-N_3 **(n = 3)**. N_3-EG3-NH_2 (0.58 g, 2.67 mmol), 4-N,N-dimethylaminopyridine (0.054 g, 0.44 mmol), N,N'-dicyclohexylcarbodiimide (0.62 g, 3.0 mmol) were mixed and dissolved in dry CH_2Cl_2 (10 mL). The mixture was cooled to 0 °C in an ice bath under N_2.

Lipoic acid (0.55 g, 2.67 mmol) dissolved in 2 mL dry CH_2Cl_2 was then slowly added through a syringe over 20 min under N_2. After addition, the reaction mixture was stirred at 0 °C for 1 h before being warmed up to gradually to RT and stirred for 24 h. After filtration and rinse the solid with $CHCl_3$, the filtrate was combined and evaporated to dryness to give an yellow oil. The crude product was purified by silica gel column chtromatgraphy using $CHCl_2$/MeOH (16:1 v/v) as eluting solvent. The desired product (**LA-EG$_3$-N$_3$**) was obtained as a yellow oil (0.86 g, 80%). R_f ($CHCl_2$/MeOH 10:1 v/v) = 0.64. ^{1}H NMR (400 MHz, $CDCl_3$) δ (ppm): 6.08 (s, 1H, amide N-*H*), 3.50-3.72 (m, 12H, C*H*$_2$s in triethylene glycol); 3.42-3.50 (m, 2H, C*H*$_2$ next to amide NH); 3.40 (t, 2 H, C*H*$_2$ next to N_3), 3.18 (m, 1 H), 3.12 (m, 1H), 2.45 (m, 1H), 2.18 (t, 2H, C*H*$_2$-C=O), 1.90 (m, 2H), 1.55-1.79 (m, 4H), 1.42-1.55 (m, 2H). FI-IR (cm^{-1}), 3450 (ν_{N-H}), 2290 (ν_{N3}), 1680 ($\nu_{C=O}$). LC-MS found: 407.1 $[M + H]^+$ and 429.1 $[M + Na]^+$.

Step 2: Synthesis of LA-PEG$_n$-NH$_2$ (n = 13 or 3)[1,7]

LA–PEG$_{13}$–N$_3$ (0.33 g, 0.389 mmol) was dissolved in dry THF (5 mL) in a 50 mL round-bottomed flask and flushed with N_2. PPh_3 (0.161 g, 0.585 mmol) was then added and the resulting solution was stirred for 30 mins, then H_2O (100 µL) was added and the reaction mixture was stirred at RT overnight under N_2. The reaction was monitored by TLC (silica gel, $CHCl_3$:MeOH = 10:1 v/v), $R_{f\,(LA–PEG13–N3)}$ = 0.62, $R_{f\,(PPh3)}$ = 0.89, $R_{f\,(LA–PEG13–NH2)}$ = 0.23). When LA-PEG$_{13}$-N$_3$ was completely reduced, the solvent was evaporated on a rotary evaporator. 5 mL EtOAc was added to dissolve the residue and the solution was transferred to a separation funnel. 1 M HCl solution (9 mL) was then slowly added with the organic layer removed. The aquesou phase was further washed with EtOAc to remove any unreacted PPh_3, LA-PEG$_{13}$-N$_3$ and formed triphenyl phosphine oxide byproduct. The aqueous layer was neutralized by Na_2CO_3 till basic (pH ~10) and then extracted by $CHCl_3$ (10 mL × 3). The organic layers were combined, dried over $MgSO_4$ and filtered. After evaporation of solvent, the desired compound (**3**) was obtained in 96% yield (0.310 g). ^{1}H NMR (500 MHz, $CDCl_3$): δ 6.63 (m, 1H), 3.5–3.7 (m), 3.37–3.4 (m, 2H), 3.07–3.28 (m, 2H), 2.87–2.99 (m, 2H), 2.7–2.8(br s, 2H), 2.44–2.48 (m, 1H), 2.17–2.23 (m, 2H), 1.86–1.96 (m, 1H), 1.57–1.73 (m, 4H), 1.39–1.51 (m, 2H).

LA-EG$_3$-NH$_2$ was synthesized in a similar way in 50% yield (0.40 g). TLC (silical gel, 10:1 $CHCl_3$:MeOH v/v), $R_{f(LA-EG3-N3)}$ = 0.62, for $R_{f(LA-EG3-NH2)}$ = 0.10. ^{1}H NMR ($CDCl_3$, δ (ppm): 6.75 (s, 1H, amide N-*H*), 3.35-3.72 (m, 14H, C*H*$_2$s in PEG units); 3.18 (m, 1 H), 3.10 (m, 1H), 2.86 (m, 2H, C*H*$_2$ next to NH_2), 2.78 (br, 2H, N*H*$_2$), 2.45 (m, 1H), 2.15-2.28 (m, 2H, C*H*$_2$-C=O), 1.80-1.92 (m, 2H), 1.55-1.75 (m, 4H), 1.40-1.55 (m, 2H). LC-MS found: 381.1 $[M + H]^+$.

Step 3: Synthesis of LA-PEG$_n$-cyclooctyne (n = 13 or 3)

For LA-PEG$_{13}$-cyclooctyne: cyclooct-1-yn-3-glycolic acid (0.0915 g, 0.50 mmol),[1] LA-PEG$_{13}$-NH$_2$ (0.493 g 0.60 mmol), DMAP (0.0124 g, 0.102 mmol) and CH_2Cl_2 (1.5 mL) were placed in a flask and flushed with N_2 for 20 mins. The reaction mixture was cooled to 0 °C in an ice bath, and a solution of DCC (0.107 g, 0.52 mmol) in DCM (1 mL) was added dropwise. The reaction mixture was stirred at 0 °C for 1 h before being warmed to RT and stirred for another ~20 h. The reaction mixture (0.50 mL) was transferred to 1.5 mL microcentrifuge tube and 1 mL EtOAc was added and centrifuged at 14 kg for 2 mins. The yellow supernatant

was transferred to a round bottom flask. The remaining precipitate was washed by EtOAc (1.5 mL) twice and centrifuged. The supernatants were combined and evaporated to dryness to give a yellow oil. TLC (silica gel, $CHCl_3$/MeOH = 10:1, v/v) $R_{f(\text{Cyclooct -1-yn-3-glycolic acid})}$ = 0.53, $R_{f\ (\text{LA-PEG600-NH2})}$ = 0.24, $R_{f\ (\text{DMAP})}$ = 0.42, $R_{f\ (\text{product})}$ = 0.67. The crude product was purified by silica gel flash column chromatography using $CHCl_3$:MeOH = 20:1 (v/v) as eluting solvent. The R_f = 0.67 fractions were collected and combined. After evaporation of solvent, the desired product (**4**, n = ~13) was obtained as a yellow oil (0.369 g, 75%). ^{1}H NMR (500 MHz, $CDCl_3$): δ(ppm) 6.3 (m, 1H), 4.0 (s, 2H), 3.8 (t, 1H), 3.5-3.6 (m), 3.37-3.4 (m, 2H), 3.07-3.2 (m, 4H), 2.4-2.5 (m, 2H), 1.7-2.3 (m, 8H), 1.57-1.73 (m, 2H), 1.4-1.7 (m, 2H), 1.0-1.4 (m, 4H).

LA-EG_3-cyclooctyne was prepared by a similar method in 33% yield as a yellow oil. TLC ($CHCl_3$/MeOH = 10:1, v/v) $R_{f\text{Cyclooct -1-yn-3-glycolic acid}}$ = 0.53, $R_{f\ \text{LA-PEG600-NH2}}$ = 0.1, $R_{f\ \text{product}}$ = 0.65. ^{1}H NMR (400 MHz, $CDCl_3$) δ (ppm): 6.85 (s, 1H, amide N-*H*), 6.10 (s, 1H, amide N-*H*), 4.24 (s, 1H), 3.70-4.10 (m, 2H, (C=O)-CH_2-O), 3.40-3.70 (m, 14H, CH_2s in EG units); 3.18 (m, 1 H), 3.10 (m, 1H), 2.45 (m, 1H), 2.10-2.30 (m, 4H), 1.00-2.00 (m, 20H). LC-MS found: 545.4 $[M + H]^+$ and 567.4 $[M + Na]^+$.

Step 4a: Synthesis of LA-PEG$_n$-Man (n = ~13 or 3)

LA-PEG_{13}-cyclooctyne (0.175 M, 114 μL, 20 μmol in ethanol) or LA-EG_3-cyclooctyne (0.144 M in ethanol, 138 μL, 19.8 μmol) was mixed with 1-azido-3,6-dioxaoct-8-yl-α-D-mannopyranoside (0.200 M in methanol, 120 μL, 24 μmol) and allowed to react at RT for 48 h for efficient conjugation. The reaction mixture was then purified by Sephadex LH20 column chromatography using methanol as eluting solvent. The desired product fractions were combined and analyzed by HR-MS. For LA-EG_3-Man: found 904.4018, calculated for $C_{38}H_{67}N_5NaO_{14}S_2$ 904.4018 ($[M+Na]^+$). A further LC-MS analysis of the LA-EG_3-Man revealed a single UV absorption band with a retention time of ~1.6 min. The corresponding MS spectrum displayed two peaks with m/z values of 882.7 and 904.7, corresponding to the $[M+H]^+$ and $[M+Na]^+$ peaks, respectively (see top panel in page 11 below).

For LA-PEG_{13}-Man, its LC-MS analysis revealed a single UV absorption band with a retention time of ~1.66 min. The corresponding MS spectrum displayed a series of species with m/z ratios separated by multiple number of 44 (the molecular weight of the EG unit): 537.7932, 595.8059, 617.8188; 639.8318, 661.8450, 683.8584, 705.8713, and 727.8832. The corresponding expected $[M+2H]^{2+}$ peaks with PEG repeat number of 9, 10, 11, 12, 13, 14, 15 and 16 were 573.7922, 595.8059, 617.8184, 639.8315, 661.8446, 683.8576, 705.8709 and 727.8840, respectively (see the figure in bottom panel, page 11 below).

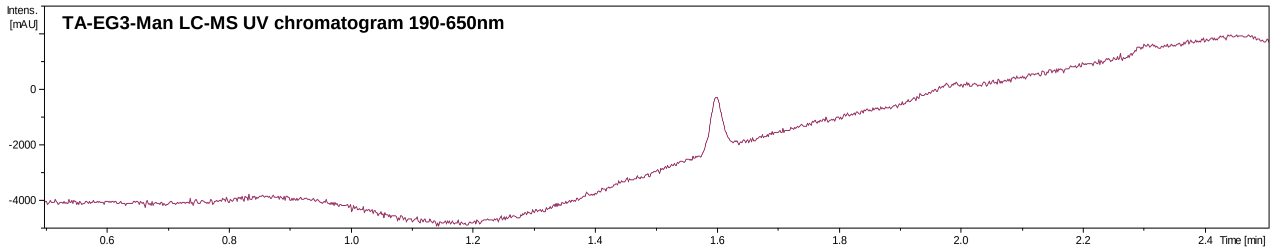

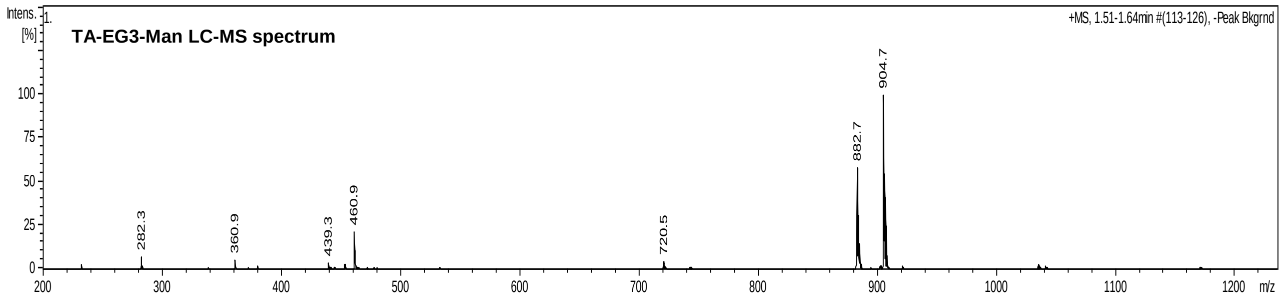

For LA-PEG$_{13}$-Man: the LC-MS spectrum gave a single absorption band with a retention time of ~1.65 min. The corresponding MS spectrum spectrum displayed a series of peaks that were separated by 44 m/z units, corresponding to different PEG chain lengths in the mixed length PEG$_{13}$ linker (see the figure below): Found: m/z peaks at 1190.9, 1235.0, 1235.0, 1323.0, 1367.0, 1411.1, 1455.1. The calculated corresponding $[M+H]^+$ peaks for LA-PEG$_n$-Man where n = 10, 11, 12, 13, 14, 15 and 16 were 1191, 1235, 1279, 1323, 1367, 1411 and 1455, respectively.

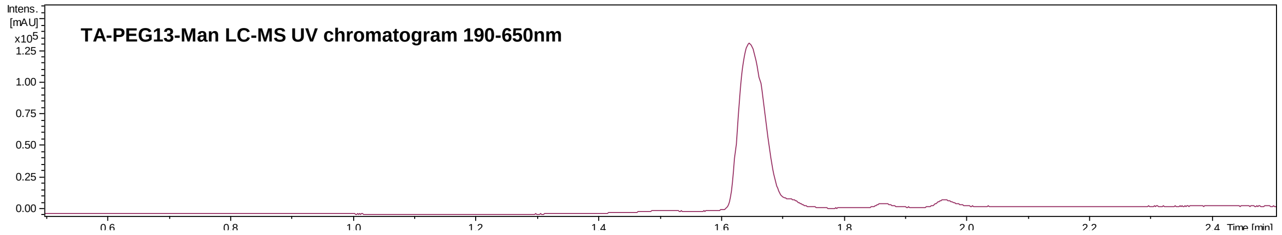

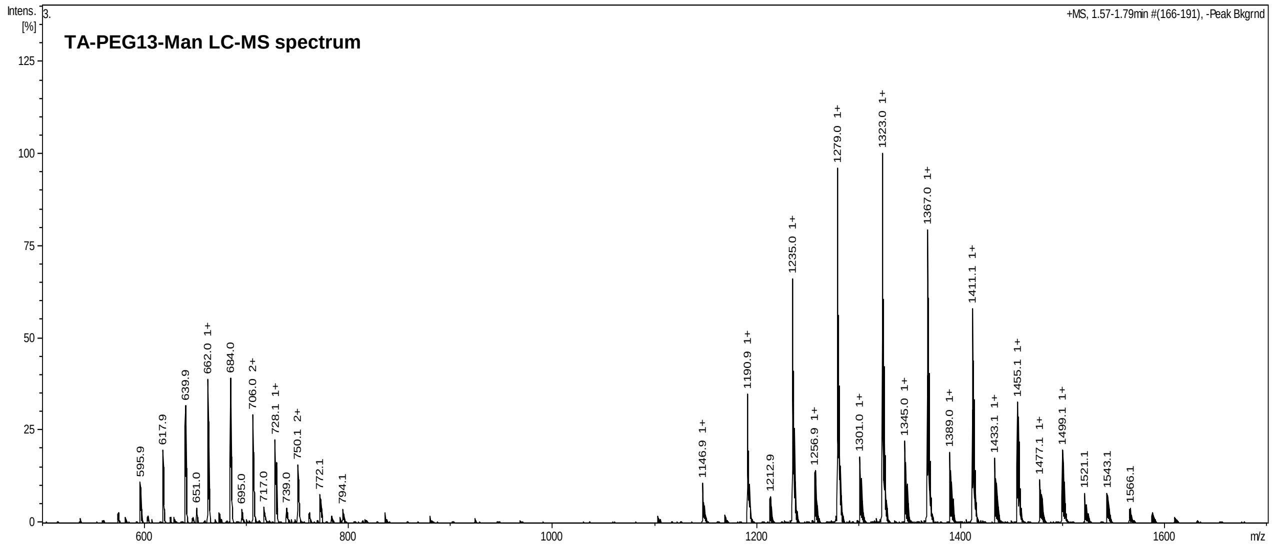

Step 4b: Synthesis of DHLA-PEG$_n$-Man (n = 13 or 3 for 8i and 8ii)

Tris(2-carboxyethyl)phosphine hydrochloride (TCEP.HCl, 1.2 mol equivalent) was dissolved in H_2O and added to the LA-PEG$_{13}$-Man or LA-EG$_3$-Man (both in EtOH) at room temperature for 30 min to reduce the

disulfide bond. After evaporation of solvent, DHLA-PEG$_{13}$-Man or DHLA-EG$_3$-Man was purified by silica gel column chromatography (chloroform:methanol, 5:1, v/v). The fractions containing reduced ligands were combined. The purified DHLA-PEG$_{13}$-Man or DHLA-EG$_3$-Man was further analysed by LC-MS. A single UV band with a retention time of ~1.6 min from the HPLC elution profile gave two MS peaks with m/z ratios of 884.6 and 906.7, corresponding to the required $[M+H]^+$ and $[M+Na]^+$ peaks (see below).

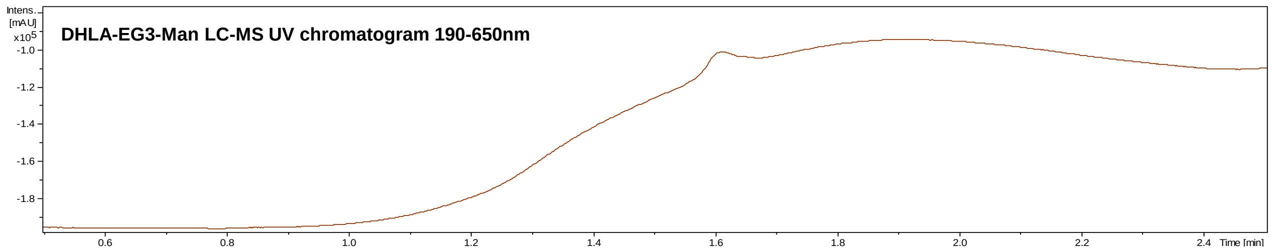

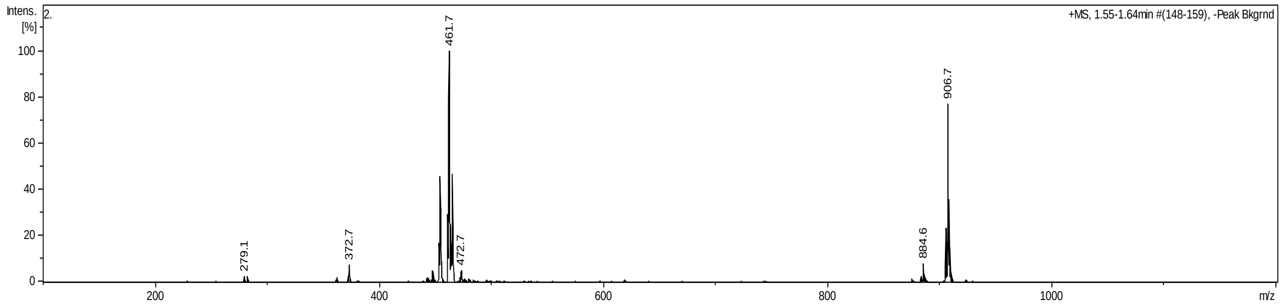

For DHLA-PEG$_{13}$-Man, a single UV absorption band from the HPLC eluting profile was found. The corresponding MS spectrum revealed a series of m/z ratios separated by 44 mass units, corresponding to the different PEG lengths of the DHLA-PEG$_{13}$-Man (see below).

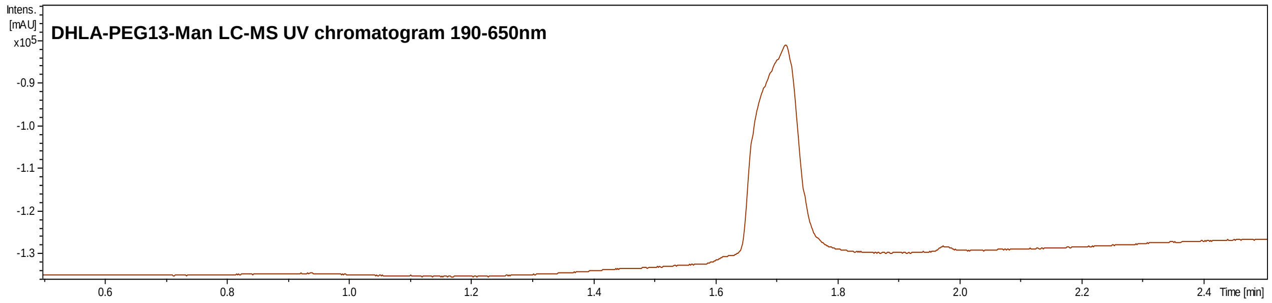

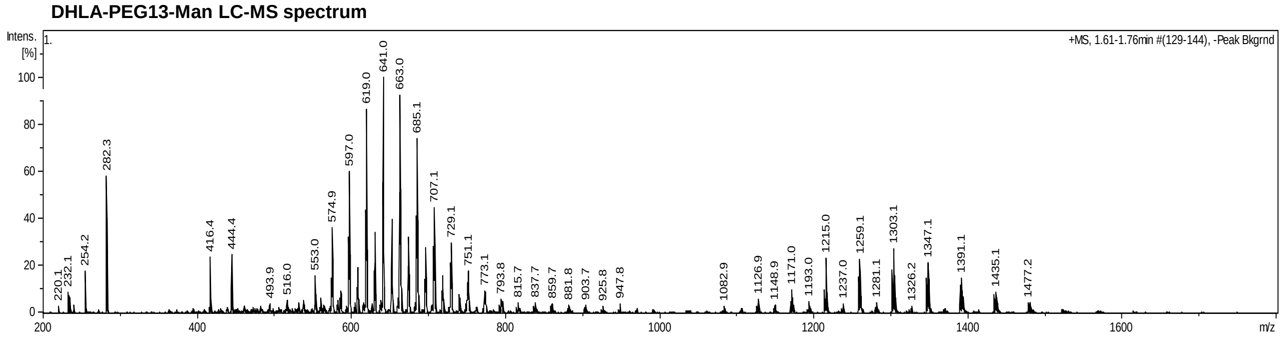

The reduced DHLA-PEG$_n$-Man ligands were concentrated and dissolved in chloroform. The concentration of mannose was determined by phenol-sulfuric acid method as described in Section A41 below.

A4) Preparation of QD-PEG13-Man and QD-EG3-Man

CdSe/ZnS core/shell QD (λ_{EM} = 560 nm, 1 nmol) in 0.2 mL toluene was precipitated by 1 mL ethanol and centrifuged at 10 × kg for 3 min.[1,3] The clear supernatant was removed and chloroform (50 µl) was added to dissolve the QD pellet. Then DHLA-PEG$_n$-Man ligand (n = 13 or 3, 500 nmol in $CHCl_3$) after deprotonation by NaOH in EtOH (0.10 M, 600 nmol in total) was added to the QD solution together with some extra methanol to make a homegenous solution ($CHCl_3$/MeOH 1:1, v/v). The reaction mixture was stirred at RT in darkness for 30 min. Hexane was then added to the reaction mixture till it became cloudy. The resulting mixture was then centrifuged at 10 × kg for 5 min to obtain QD-PEG$_{13}$-Man/QD-EG$_3$-Man pellet. After removal of clear supernatant (checked by UV to ensure no QD fluorescence), the pellet was dissolved in 100 µL of pure H_2O. The QD conjugate solution was then transferred to a 30 KD MWCO spin column and washed with H_2O (100 µL × 3) to remove any unbound free ligands. The QD stock concentration was determined by measuring its absorbance at 546 nm using extinction coefficient of 1.3×10^5 $M^{-1}\cdot cm^{-1}$.[1,3]

A41) Determination of mannose loading on the QDs[8]

Mannose amount was determined by phenol-sulfuric acid method. D-mannose stock (100 mg/mL in H_2O) was diluted 100 times to give 1 mg/mL stock in H_2O. Different amounts of stock solution were then mixed with 5% phenol in H_2O and sulphuric-acid to generate a calibration curve as follows:

To a solution of D-mannose in H_2O (125 µl) in a 5 mL-glass vial was added 125 µL of 5% phenol in H_2O followed by 625 µL of concentrated H_2SO_4. The mixture was vertexed and allowed to stand at RT for 30 min. The absorbance of the solution at 490 nm was measured against a blank pure water control solution to generate a calibration curve.[8]

Unconjugated DHLA-PEGn-Man ligands collected from hexane precipitation supernatant (after removal of organic solvent and dissolving in H_2O) and spin column filtrate were combined to make a total volume of 1300 µL for DHLA-PEG$_{13}$-Man and 1430 µL for DHLA-EG$_3$-Man. 125µL of each solution was then mixed with 125 µL of phenol and 625 µL of H_2SO_4 as above to determine the amounts of unconjugated mannose ligand. The dilution factors were then corrected to calculate the total amount of unconjugated mannose ligand. Assuming the difference between the amounts of ligand added and unconjugated were those that have bound to the QD,[1,3] the average number of mannose molecules conjugated to each QD was then calculated as 173±37 and 327±74 for QD-PEG$_{13}$-Man and QD-EG$_3$-Man, respectively. A lower mannose valency in the former is likely due to a relatively long PEG chain that may sterically limit very high ligand packing on the QD surface, leading to a lower ligand density.

A42) Transmission electron microscopy (TEM) and Dynamic light scattering (DLS)

TEM imaging was performed using a Philips CM200 transmission electron microscope by depositing a drop of the quantum dot solution onto carbon-coated grid as previously described.[2]

DLS was performed using a Zetasizer NanoZS (Malvern) using a laser wavelength of 633 nm in disposable polystyrene cuvettes. QD conjugates were in binding buffer (100 mM NaCl, 20 mM HEPES 7.8, 10 mM

$CaCl_2$ and 10 μg/ml His_6-Cys peptide) and measured without filtration. Readings of 10 scans were taken in triplcate and the average values were calculated.[2]

A43) Calculation of inter-mannose distance (X)[9]

For QD of radius *r*, each conjugated with N number of ligands, footprint for each ligands:

$$k = \frac{4\pi r^2}{N} \qquad (1)$$

The average deflection angle for each ligand can be calculated by:

$$\theta = \frac{2\times 180\times\sqrt{\frac{k}{\pi}}}{r\pi} = \frac{229.3}{\sqrt{N}} \qquad (2)$$

Where θ for QD-EG_3-Man and QD-PEG_{13}-Man (N = 327 and 173) are calculated as 12.68 and 17.43^{o} respectively.

The inter-mannose distance on the QD surface (X) can be calucated *via*:

$$X = 2\times R\times \sin(\frac{\theta}{2}) \qquad (3)$$

where R is hydrodynamic radius of the QD-Man conjugate as measured by DLS (SI Fig. S1), R = 8.9/2 = 4.45 nm for QD-EG_3-Man and 9.6/2 = 4.8 nm for QD-PEG_{13}-Man. Using the above paramters, the inter-Man distance:

For QD-EG_3-Man: X = 2 × 4.45 sin(12.68/2) = 0.98 nm

For QD-PEG_{13}-Man: X = 2 × 4.8 sin(17.43/2) = 1.46 nm.

A5) Protein Production, Labeling and FRET Analysis

A51: protein production[10,11]

Cysteine was introduced into the cDNAs encoding extracellular segment of DC-SIGN to replace residue Q274 or DC-SIGNR to replace R287 for site-specific dye labelling as indicated in the extracellular segment amino acid alignment of DC-SIGN/R below (star indicating Q274 in DC-SIGN and R287 in DC-SIGNR)

The mutagenesis was carried out by using synthetic DNA restriction fragments to replace the corresponding wild-type sequences. Standard recombinant DNA techniques were used throughout these experiments. The integrity and successful mutation of the cloned fragments were confirmed by DNA sequencing.

```
DC-SIGN:  KVPSSISQEQSRQDAIYQNLTQLKAAVGELSEKSKLQEIYQELTQLKAAVGELPEKSKLQEIYQELTRLKAA
DC-SIGNR: KVPSSLSQEQSEQDAIYQNLTQLKAAVGELSEKSKLQEIYQELTQLKAAVGELPEKSKLQEIYQELTRLKAA

DC-SIGN:  VGELPEKSKLQEIYQELTWLKAAVGELPEKSKMQEIYQELTRLKAAVGELPEKSKQQEIYQELTRLKAAVGEL
DC-SIGNR: VGELPEKSKLQEIYQELTRLKAAVGELPEKSKLQEIYQELTRLKAAVGELPEKSKLQEIYQELTELKAAVGEL

                                                        ↱ CRD
                                                                          *
DC-SIGN:  PEKSKQQEIYQELTRLKAAVGELPEKSKQQEIYQELTQLKAAVERLCHPCPWEWTFFQGNCYFMSNSQRNWHD
DC-SIGNR: PEKSKLQEIYQELTQLKAAVGELPDQSKQQQIYQELTDLKTAFERLCRHCPKDWTFFQGNCYFMSNSQRNWHD
                                                                           *

DC-SIGN:  SITACKEVGAQLVVIKSAEEQNFLQLQSSRSNRFTWMGLSDLNQEGTWQWVDGSPLLPSFKQYWNRGEPNNVG
DC-SIGNR: SVTACQEVRAQLVVIKTAEEQNFLQLQTSRSNRFSWMGLSDLNQEGTWQWVDGSPLSPSFQRYWNSGEPNNSG

DC-SIGN:  EEDCAEFSGNGWNDDKCNLAKFWICKKSAASCSRDEEQFLSPAPATPNPPPA
DC-SIGNR: NEDCAEFSGSGWNDNRCDVDNYWICKKPAA-CFRDE
```

Extracellular segments (sequence as above) which were known to form stable homotetramers and faithfully retaining their glycan binding properties[4] were expressed in *E.coli* and purified by Man-Sepharose affinity column as described previously.[10,11] The monomeric DC-SIGN and DC-SIGNR CRDs were also constructed and purified as described previously.[10,11] The prufity of the recombinant proteins was confirmed by gel electrophresis as shown below:

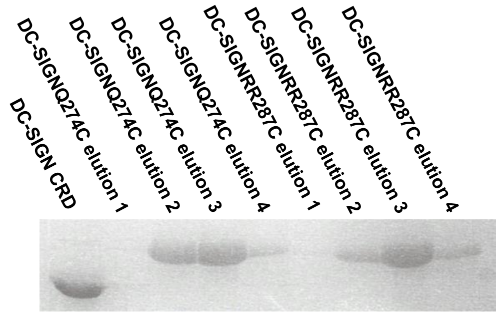

SDS-polyacrylamide gel electrophoresis of DC-SIGN Q274C, DC-SIGNR R287C following purification by Man-Sepharose affinity chromatography. The gel (17.5% polyacrylamide) was stained with coomassie blue.

A52: Protein labelling[12]

For labelling DC-SIGNQ274C and DC-SIGNRR287C, proteins were first bound to a mannose-Sepharose column and thoroghly washed with HEPES buffer (20 mM HEPES pH 7.4, 100 mM NaCl, 25 mM $CaCl_2$) to remove free and weakly bound proteins (*ca.* 5-10 column volumes). The bound proteins were then eluted out by changing to an EDTA containing eluting buffer (20 mM HEPES pH7.4, 100 mM NaCl, 2.5 mM EDTA) because of the Ca^{2+}-dependent DC-SIGN/R-sugar binding. $CaCl_2$ was added to the eluted protein fractions to reach a final concentration of 25 mM. 1.5-2 mol equivalent of Atto594-maleimide in DMSO was added to the proteins (with total DMSO volume < 5% the reaction mixture). The mixture was wrapped in a foil and rocked in the darkness for 1 h at RT and then at 4°C overnight. The labeled protein was purified by mannose-Sepharose affinity column, washed first with washing buffer (20 mM HEPES pH 7.8, 100 mM NaCl, 10 mM

$CaCl_2$) to remove any non- or weakly binding species and then eluted out by eluting buffer (20 mM HEPES pH 7.8, 100 mM NaCl, 2.5 mM EDTA) because of the Ca^{2+}-dependent binding property. The eluted coloured fractions (containing labeled proteins) were collected and $CaCl_2$ was added to a reach final concentration of 10 mM. The labelling efficiency was determined by measuring the absorbance at 280 nm for protein and at 608 nm for Atto594 dye on a NanoDrop 2000 Spectrophotometer. The protein and dye concentrations were calculated by using extinction coefficients of 2.81 $\times 10^5$ $M^{-1}\cdot cm^{-1}$ for DC-SIGN, 2.43 $\times 10^5$ $M^{-1}\cdot cm^{-1}$ for DC-SIGNR at 280 nm, and 1.2 $\times$ 10^5 $M^{-1}.cm^{-1}$ at 605 nm for the dye. Dye absorption contribution at 280 nm was corrected by a correcting factor of 0.51 as given in supplier's protocol. The average labeling of Atto594 dye on each CRD was found to be 0.82 for DC-SIGNQ274C and 0.85 for DC-SIGNRR287C.[12]

The monomeric DC-SIGN and DC-SIGNR CRDs were labeled by with 3-4 mol equivalent Atto594-NHS ester in 20 mM HEPES, pH 8.3, 150 mM NaCl and 100 mM $CaCl_2$ over night at 4°C. Protein concentration was estimated using extinction coefficient of 5.3 $\times 10^4$ $M^{-1}\cdot cm^{-1}$ at 280 nm and dye extinction coefficient at 605 nm of 1.2 $\times 10^5$ $M^{-1}.cm^{-1}$. The average labelling of Atto594 dye on each CRD was found to be 1.5.

A53: DC-SIGN-QD binding, sugar competition, calcium dependent and temperature dependent binding assay

Fluorescence spectra were recorded on a FluoroMax-3 spectrofluorimeter (λ_{EX} = 450 nm, corresponding to the minimum absorption of the Atto594 to minimize the direct excitation background) over 480-800 nm with excitation and emission slit widths of 5 nm. All measurements were done in the binding buffer (20 mM HEPES pH 7.8, 100 mM NaCl, 10 mM $CaCl_2$) containing 10 µg/mL of a His_6-Cys peptide which was found to increase the QD stability and reduce non-specific adsorption.[1] The final QD concetration (C_{QD}) was all 40 nM. The labeled proteins were mixed with the QD at room temperature for 20 min before fluorescence spectra were recorded. Binding of labeled monomeric DC-SIGN CRD or DC-SIGNR CRD with the QD-mannose conjugate was performed in the same way as described above.

For calcium dependent binding, the labeled DC-SIGN protein in buffer (20 mM HEPES, 100 mM NaCl, 2.5 mM EDTA, pH 7.8) was dialysed extensively againest a calcium free buffer (20 mM HEPES, 100 mM NaCl, pH 7.8) to remove EDTA. Then the labeled protein (final $C_{protein}$ = 0.38 µM) was mixed with QD-Mannose (final C_{QD} = 40 nM) in calcium free buffer containing His_6-Cys (10 µg/mL). Different amount of calcium was then added to the mixture and incubated at room temperature for 20 min before measurement.

For sugar competition assay, mannose (Man) or galactose (Gal) stock was first made in the binding buffer. Different amount of Man or Gal was mixed with the labeled DC-SIGN (final $C_{protein}$ = 0.5 µM) first before adding to the QD-mannose conjugates (final C_{QD} = 40 nM). The mixture was incubated at room temperature for 20 min before measurement.

For temperature dependent binding assay, the temperature of the resulting solution was controlled by a water bath connected to the fluorimeter sample holder. Each sample mixture was incubated at an designated temperature for 20 min before measurement was taken. Acceptor direct excitation background corrected fluorescence spectra of DC-SIGN (0.5 µM) binding to QD-PEG_{13}-Man or QD-EG_3-Man (C_{QD} = 40 nM) at

different temperature were shown in Figure S7. The apparent FRET ratio (I_{626}/I_{554}) was used to calculate the coresponding K_D at different temperature (see data analysis below).

A54: Data Analysis:[13,14]

For all of the FRET related measurements, the raw fluorescence spectra of labeled protein + QD samples were substracted by the fluorescence spectra of the corresponding dye-labeled protein at same concentration only under identifical conditions to correct the background arising from the acceptor dye direct excitation.

(a) Binding affinity evaluation. Fluorescence intensities at 554 nm (QD emitting peak wavelength, all final C_{QD} = 40 nM) and 626 nm (for Atto-594 dye FRET peak wavelength) were used to calculate the apparent FRET ratio, I_{626}/I_{554}. The I_{626}/I_{554} FRET ratio was ploted against the protein concentration and plots were fitted to a Hill's equation using the equation below (OriginPro8.6, OriginLab):[13]

$$I_{626}/I_{554} = (Max \times [C]^n)/(K_D{}^n + [C]^n) \qquad (1)$$

Where Max is the saturate FRET ratio, K_D is the protein concentration at half saturate binding (equivalent to apparent dissociation constant, K_D), [C] is the total protein concentration and n is Hill's coefficient. The data were fitted first to give estimated values of $Max_{(0)}$, $K_{D(0)}$ and $n_{(0)}$. Iterative fittings were then applied to improve the fitting by using n as a fitting parameter: n was changed manually in the range of $n_{(0)} \pm 0.5$ in a step size of 0.2. The best fit was evaluated by the resulting R square value (R^2) as below (Table S1).

(b) Evaluation of inhibition constant K_I. Direct excitation background corrected fluorescence spectra were used to derive the apparent FRET ratio of I_{626}/I_{554} as above. It was then plotted against inhibitor (mannose) concnetration. The data were fitted to a simple first order equation as below:

$$F = K_I/(K_I + [mannose]) \qquad (2)$$

$$F = \frac{(I_{626}/I_{554})_m}{(I_{626}/I_{554})_0} \qquad (3)$$

Where F is the normalised fraction of dye-labelled-protein bound to QD, $(I_{626}/I_{554})_0$ is the FRET ratio of dye-labeled protein + QD in the absence of competing mannose, and $(I_{626}/I_{554})_m$ is the apparent FRET ratio in the prescence of mannose. K_I is the the concentration of mannose that yields 50% inhibition of QD binding (FRET ratio drops to 50% of the initial level) and [mannose] is the total concentration of mannose.

(c) Evaluation of binding enthalpy and entropy.[14] The apparent FRET ratio (I_{626}/I_{554}) were obtained after correction of dye direct excitation background as above. K_D at each temperature was calculated *via* equation (1) using the Max and n values obtained from the best fit at 25 °C (Table S1) and given in Table S2.

A55) Correlation between FRET ratio and QD bond proteins[1]

For a single QD donor which is in FRET interaction with ***N*** identical acceptors (*e.g.* under idential QD-dye distance *r*), the FRET efficiency, *E*, can be given in the following equation:

$$E = N\times R_0^6/[N\times R_0^6 + r^6] = 1/[1 + r^6/(N\times R_0^6)] \qquad (4)$$

where R_0 is the Förster radius of the QD-single dye FRET pair and r is donor-acceptor distance. E can also be measured *via* the enhanced acceptor emission by the following equation:

$$E = I_{Dye}/[I_{Dye} + \gamma \times I_{QD}] = 1/[1 + \gamma \times I_{QD}/I_{Dye}] \quad (5)$$

Where γ is a correcting factor for the different dye and QD fluorescence quantum yield. Assuming that the shape of the QD and dye fluorescence spectra are independent of their intensity, then the integrated I_{QD}/I_{Dye} ratio should be linearly propotional to the peak intensity ratio, *e.g.* $I_{QD}/I_{Dye} = \alpha\ I_{554}/I_{626}$ (where α is a correction factor between the integrated and peak intensity ratio, which is found to be 1.50 here).

The combination of equations (4) and (5) gives the following equation:

$$1/[1 + \gamma \times I_{QD}/I_{Dye}] = 1/[1 + \gamma \times \alpha \times I_{554}/I_{626}] = 1/[1 + r^6/(N \times R_0^6)] \quad (6)$$

This yields the following relationship:

$$\gamma \times \alpha \times I_{554}/I_{626} = r^6/(N \times R_0^6) \quad (7)$$

Hence $$\mathbf{I_{626}/I_{554} = N\ [\gamma \times \alpha \times (R_0/r)^6]} \quad (8)$$

Where γ, α and R_0 are all constant values. This equation shows that the apparent FRET ratio I_{626}/I_{554} should increase linearly with $\boldsymbol{N}$, the number of acceptors (proteins) bound to each QD if all of the proteins were bound to the QD in the same distance r.

A6) Viral Inhibition Assays[15]

Effects of the QD-EG_3-Man in inhibiting pseudo-Ebola infection of 293T cells were assessed by using our established procedures.[15] Brifely, 293T cells seeded in 96-well plates were transfected with plasmids encoding DC-SIGN or DC-SIGNR or control transfected with empty plasmid (pcDNA). The cells were washed at 6 h post transfection and further cultivated at 37°C, 5% CO_2 in Dulbecco's modified eagle medium (DMEM) containing 10% fetal bovine serum (FBS). At 48 h post transfection, the cells were then twice exposed to different concentrations of the QD-EG_3-Man inhibitor in DMEM supplemented with 10% FBS for 30 min in a total volume of 50 µl. Thereafter, after washing and disposal of the QD-containing medium, the cells were inoculated with 50 µl of MLV retroviral vectors (encoding luciferase gene) bearing either the Ebola virus glycoprotein (EBOV-GP, which binds specifically to DC-SIGN/R to facilitate vector cell entry) or the vesicular stomatitis virus glycoprotein (VSV-G, which does not bind to DC-SIGN/R and hence does not drive vector cell entry). The specific binding between the QD-EG_3-Man and 293T cell surface DC-SIGN/Rs would block and inhibit their binding to MLV-EBOV-GP surface glycans, reducing the vectors' tranduction efficiency and hence cellular luciferase activity. Otherwise, the cellular luciferase activity would be unaffected. At 6 h post inoculation, 100 µl of fresh DMEM culture medium was added and the cells incubated for 72 h. After that the cells were wahsed and the luciferase activities in the cell lysates were determined using a commercially available kit (PJK), following the manufacturer's instructions as described in our previous publication.[15]

Table S1. Binding parameters for the DC-SIGN-QD-PEG_n-Man interactions derived from the fits of Fig. 2F

QD-nanoprobe + Receptor	Apparent K_D (µM)	Max I_{626}/I_{554}	n	Adj. R-square R^2
DC-SIGN + QD-PEG_{13}-Man	0.60±0.10	3.3±0.2	1.7	0.9989
DC-SIGN + QD-EG_3-Man	0.31±0.03	17.0±0.6	1.6	0.99104

Table S2. Calculated thermodynamic parameters of DC-SIGN binding to QD-PEG_n-Man conjugates.

QD samples	Measured FRET ratio (I_{626}/I_{554})			Calculated K_D (µM)		
	25°C	31°C	37°C	25°C	31°C	37°C
QD-PEG_{13}-Man	1.43±0.02	0.95±0.02	0.66±0.01	0.56±0.01	0.86±0.01	1.15±0.01
QD-EG_3-Man	16.7±0.2	14.8±0.2	13.2±0.5	0.12±0.01	0.20±0.01	0.28±0.03

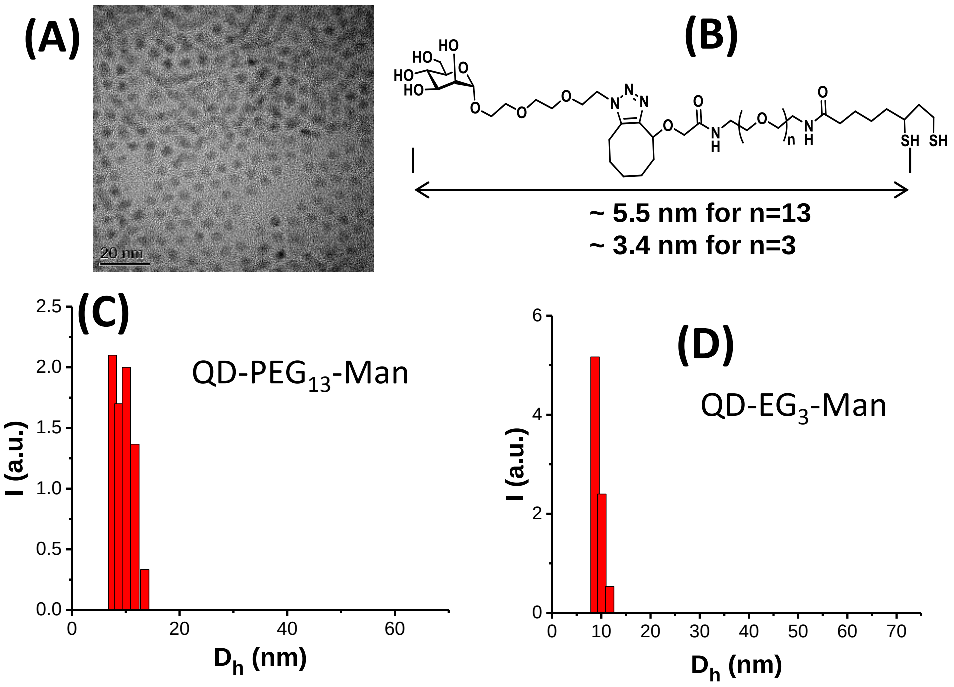

Figure S1. (**A**) Representative TEM image of the CdSe/ZnS core/shell QD used in this study. The QD core size is found to be ~4.2 nm.

(**B**) Schematic showing the molecular lengths of the naturally extended DHLA-PEG$_{13}$-Man (~5.5 nm) and DHLA-EG$_3$-Man (~3.4 nm) ligands estimated by adding the PEG linker Flory radius (**F**, where F = n × 0.35 × 0.6 nm, n is the number of PEG units, see Jokerst, J. V. *et al. Nanomedicine*, **2011**, 6, 715) and other parts of the extended ligand structures using the standard bond-length and angle for the C-C, C-O, C-N and O-H bonds. The fully stretched molecular lengths for DHLA-PEG$_{13}$-Man and DHLA-EG$_3$-Man ligands with all the PEG units fully extended are estmiated as 4.4 and 7.9 nm, respectively.

(C, D) Representative hydrodynamic diameter (D_h) distribution hostograms of the QD-PEG$_{13}$-Man (**C**) and QD-EG$_3$-Man (**D**) measured by dynamic light scattering, giving a D_h of ~9.6 and ~8.9 nm, respectively. These measured D_hs were smaller than those estimated from the sum of the QD core plus twice the natural extended ligand lengths (*ca.* 15.2 and 11.0 nm, respectively), suggesting that the natural conformation of the ligands were not extended.

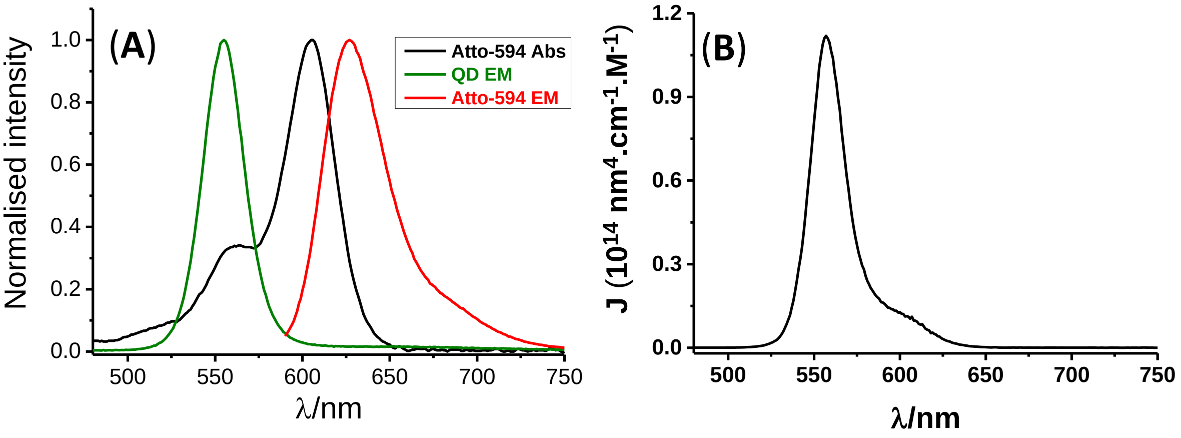

Figure S2. (**A**) Normalised fluorescence spectra of the QD (**green**) and Atto-594 (**red**) as well as the Atto-594 absorption spectrum (**black**). (**B**) Spectral overlap function ($J_{(\lambda)}$) of the QD-Atto-594 FRET pair. It is calculated *via* the following equation:

$$J_{(\lambda)} = \frac{\int PL_{D(\lambda)}\, \varepsilon_{A(\lambda)}\, \lambda^4\, d\lambda}{\int PL_{D(\lambda)} d\lambda}$$

Where $PL_{D(\lambda)}$ is the normalised QD fluorescence intensity at λ; $\varepsilon_{A(\lambda)}$ is the acceptor absorption coefficient at λ. The integral of the spectral overlap for the QD-Atto-594 pair: $I = 3.47 \times 10^{15}$ ($nm^4.cm^{-1}.M^{-1}$)

Using Rhodamine 6G in ethanol (QY = 95%, λ_{EX} = 480 nm) as reference standard, the quantum yields, Q_{QY}, of the QD-PEG$_{13}$-Man and QD-EG$_3$-Man were determined as 33% and 24%, respectively. Assuming a refractive index of 1.45 for the medium and random orientation of the dipoles (K^2 = 2/3), then the Förster radius (R_0, given in the unit of Å) of the above QD-dye FRET pair (at 1:1 molar ratio) can be calculated *via* the following equation:[1,16]

$R_0 = (8.79 \times 10^{-5}\, n_r^{-4} \times Q_{QY} \times K^2 \times I)^{1/6}$

For QD-EG$_3$-Man,

$R_0 = (8.79 \times 10^{-5} \times 1.45^{-4} \times 0.24 \times (2/3) \times 3.47 \times 10^{15})^{1/6}$

= 47 Å

= 4.7 nm

For QD-PEG$_{13}$-Man,

$R_0 = (8.79 \times 10^{-5} \times 1.45^{-4} \times 0.33 \times (2/3) \times 3.47 \times 10^{15})^{1/6}$

= 50 Å

= 5.0 nm

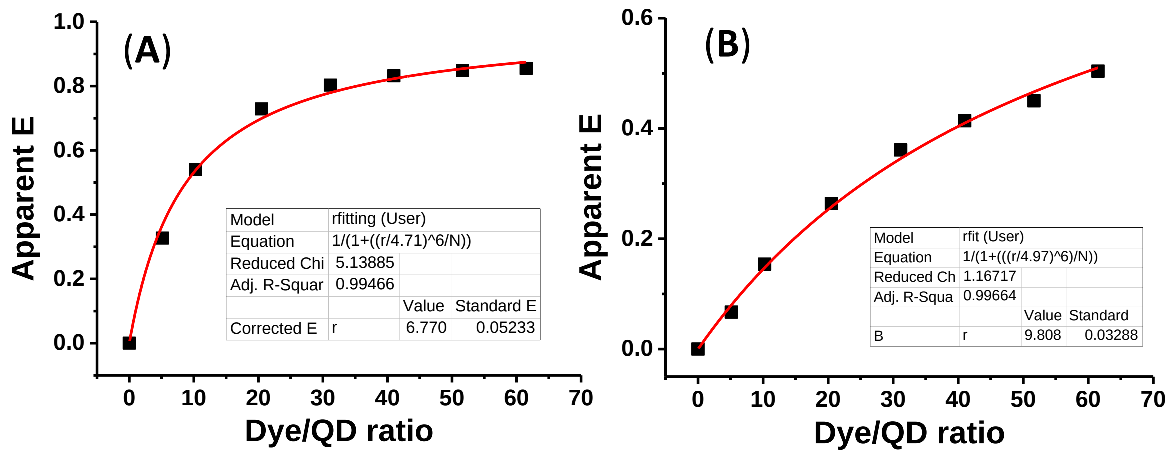

Figure S3: The relationships between apparent FRET efficiency (*E*) as a function of Dye/QD molar ratio (*N*) for DC-SIGN binding to QD-EG_3-Man (**A**) and QD-PEG_{13}-Man (**B**). The data were fitted by a single QD donor FRET to multiple identical accepters:

$$E = N\times R_0^6/(r^6 + N\times R_0^6) = 1/[1 + r^6/(N\times R_0^6)]$$

Where r is the average QD centre-dye distance and R_0 is the Förster radius (4.71 nm for QD-EG_3-Man and 4.97 nm for QD-PEG_{13}-Man). The apparent FRET efficiency E was obtained by acceptor enhancement using the following equation:

$$E = I_{Dye}/[I_{Dye} + \gamma \times I_{QD}] = 1/[1 + \gamma \times I_{QD}/I_{Dye}]$$

Where I_{Dye} and I_{QD} are the integrated acceptor and QD fluorescence intensities and γ is a correction factor for the different quantum yield (η) of the dye (0.85) and the QD, where $\gamma = \eta_{Dye}/\eta_{QD}$. The ηs are 0.24 and 0.33 for QD-EG_3-Man and QD-PEG_{13}-Man, yielding γ = 3.54 or 2.58 for the Atto594/QD-EG_3-Man or QD-PEG_{13}-Man FRET pair, respectively. The I_{Dye} and I_{QD} values are obtained from the acceptor direct excitation background corrected fluorescence spectra shown in Figures 2A and 2B. Only DC-SIGN concentration of up to 0.75 μM was used in the data fitting (before the I_{626}/I_{554} ratio reaching saturation)

The Dye/QD molar ratio was calculated from the DC-SIGN/QD molar ratio used in each measurement (C_{QD} = 40 nM), where each DC-SIGN tetramer contains an average of 3.28 Atto594 dyes (the dye labeling efficiency on each CRD monomer was determined as 0.82).

Both curves yielded excellent fits with the single QD donor FRET with multiple acceptor model (R^2 = 0.995 and 0.997, respectively). The best fits gave an average QD centre-dye distance (r) of ~6.8 and ~9.8 nm for DC-SIGN binding to QD-EG_3-Man and QD-PEG_{13}-Man, respectively.

Desipte similar D_h values for QD-PEG_{13}-Man and QD-EG_3-Man (9.6 *v.s.* 8.9 nm) prior to DC-SIGN binding, the considerably longer QD-dye distance r observed for QD-PEG_{13}-Man over QD-EG_3-Man (9.8 *v.s.* 6.8 nm) indicated that binding with DC-SIGN changed the conformation of the ligands on the QD surface. The r values (9.8 and 6.8 nm) roughly equaled to the QD core radius plus respective fully-stretched ligand length

(calculated as 10.0 and 6.5 nm for QD-PEG$_{13}$-Man and QD-EG$_3$-Man respectively), suggesting that the ligands were stretched upon multivalent binding with DC-SIGN.

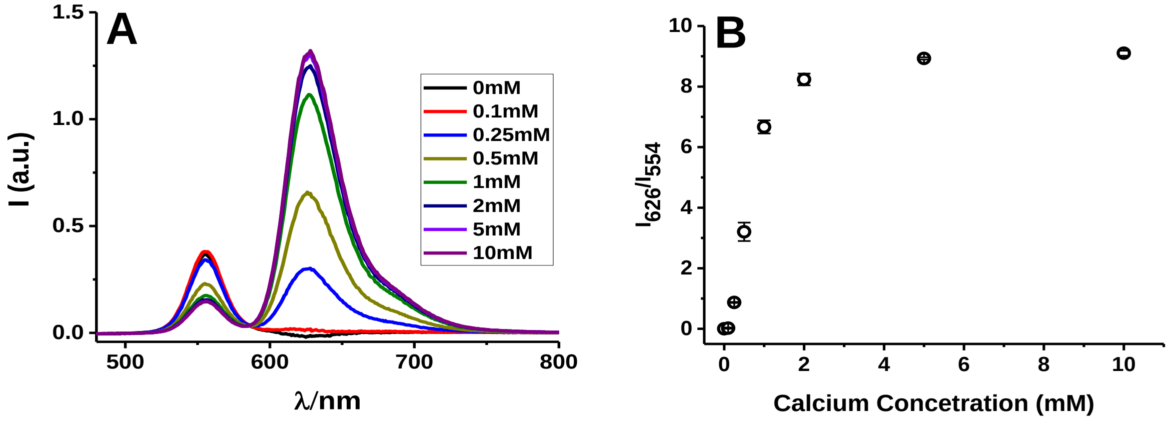

Figure S4. Ca^{2+}-dependent DC-SIGN-QD-EG$_3$-Man binding. (**A**) Dye-direct excitation background corrected fluorescence spectra showing the binding of DC-SIGN (0.38 μM) with 40 nM of QD-EG$_3$-Man in the HEPES buffer with different amounts of Ca^{2+}. (**B**) Relationship between the apparent FRET ratio (I_{626}/I_{554}) and Ca^{2+} concentration.

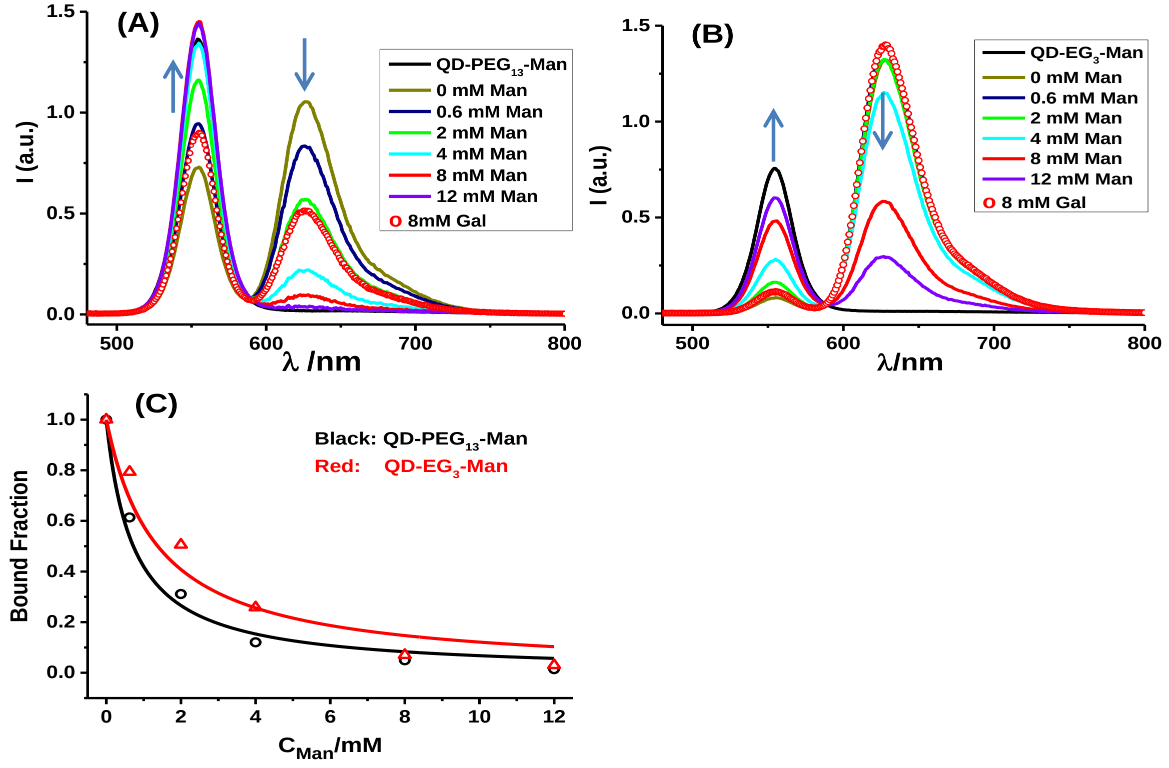

Figure S5. Inhibition curves for QD-Man binding by DC-SIGN in the presence of mannose and galactose. Fluorescence spectra showing the binding of DC-SIGN (0.5 μM) with 40 nM of QD-PEG$_{13}$-Man (**A**) or QD-EG$_3$-Man (**B**) in the presence of different amounts of free mannose or 8 mM galactose. (**C**) Relationship between the normalized bound receptor fractions as a function of mannose concentration. The relative binding values is normalized by their respective apparent FRET ratios (I_{626}/I_{554}) in the absence of any added free mannose.

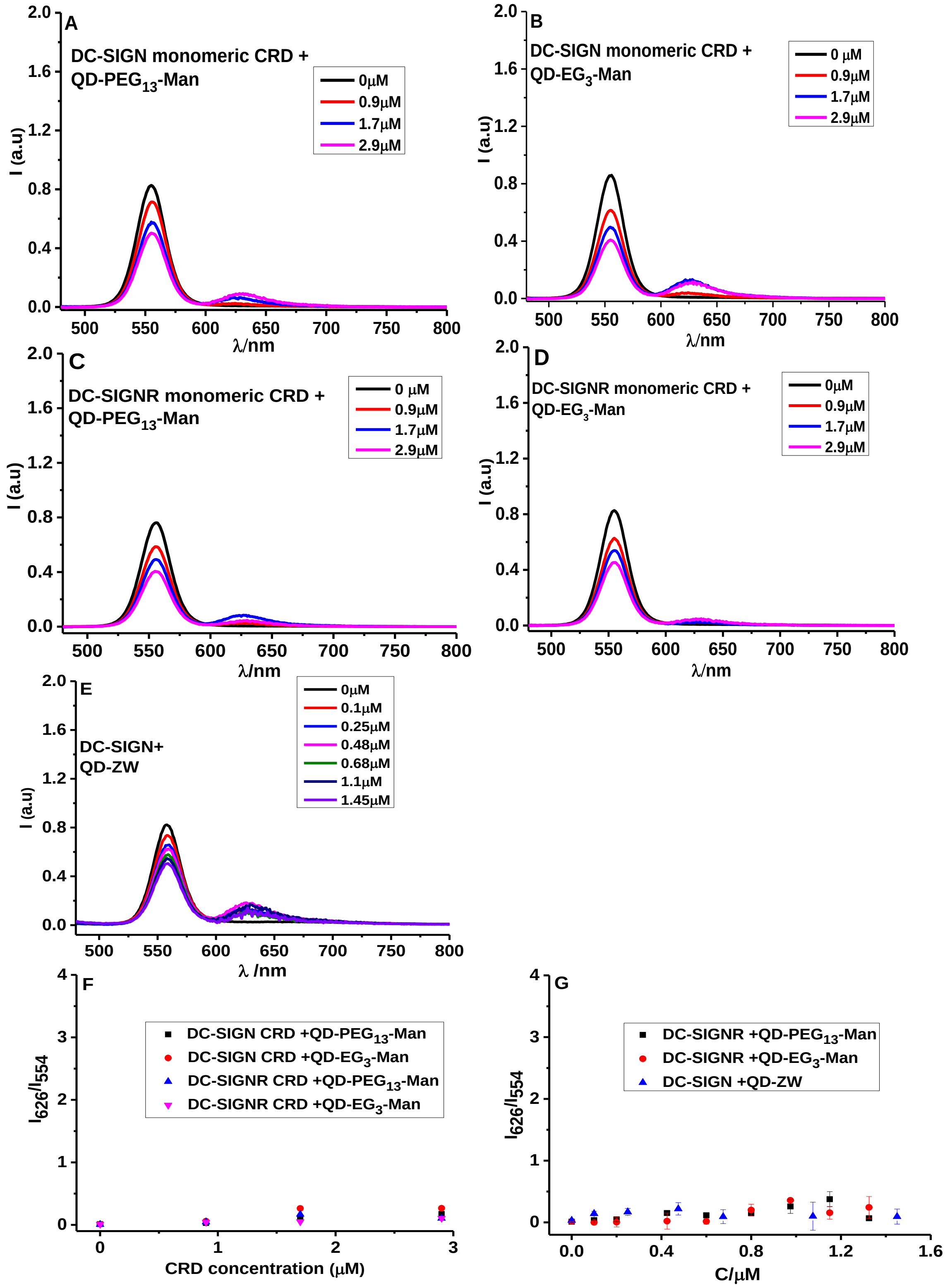

Figure S6: Background corrected fluorescence spectra of the monomieric DC-SIGN or DC-SIGNR CRD binding to QD-PEG$_{13}$-Man or QD-EG$_3$-Man (**A-D**) and tetrameric DC-SIGN binding to QD-zwitterion (QD-ZW) (**E**). Relationship between apparent FRET ratio (I_{626}/I_{554}) and monomeric CRD protein concentration for the QD-PEG$_{13}$-Man and QD-EG$_3$-Man (**F**); and tetrameric DC-SIGN for QD-ZW, tetrameric DC-SIGNR for the QD-PEG$_{13}$-Man and QD-EG$_3$-Man (**G**). The final C_{QD}s were 40 nM.

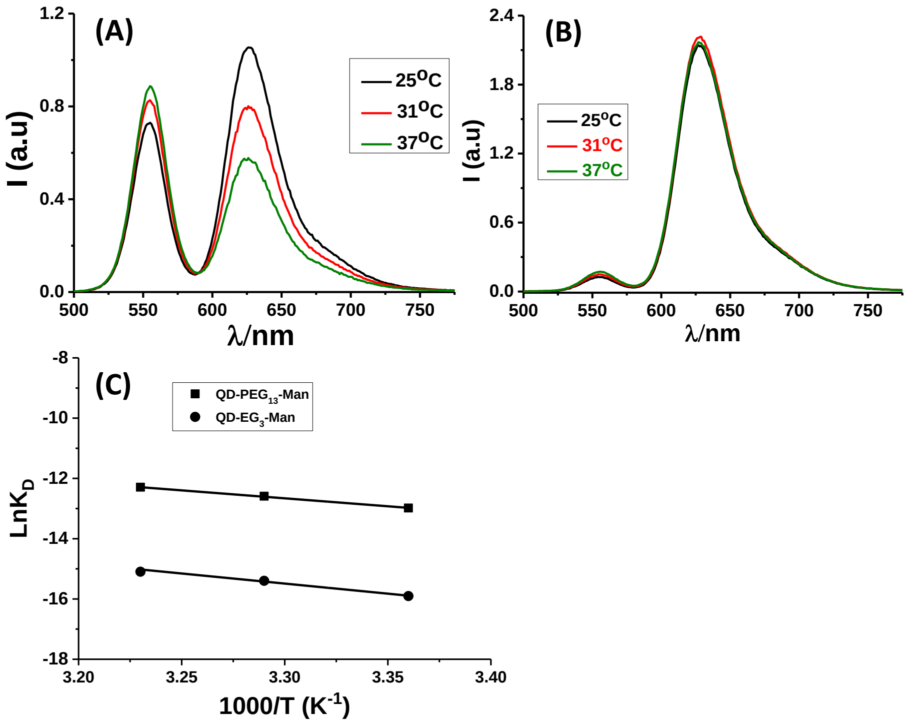

Figure S7. Tmeperature dependent DC-SIGN-QD-Man binding (**A, B**) Dye direct excitation background corrected fluorescenec spectra of DC-SIGN binding to QD-PEG13-Man (**A**) and QD-EG3-Man (**B**) at different temperatures. (C) The corresponding Arrhenius plots of DC-SIGN binding with QD-EPG13-Man (solid squares) and QD-EG3-Man (solid circles).

References

[1] Zhang, H.; Guo, Y.; Feng, G.; Zhou, D. *Nanoscale* **2013**, *5*, 10307.

[2] Song, L.; Ho, V.B.H.; Chen, C.; Yang, Z.; Liu, D.; Chen, R.; Zhou, D. *Adv. Healthcare Mater*. **2013**, 3, 275.

[3] Zhou, D.; Ying, L.; Hong, X.; Hall, E. A.; Abell, C.; Klenerman, D. *Langmuir*, **2008**, *24*, 1659.

[4] Zhan, N.; Mattoussi, H. *ACS Appl. Mater. Interfaces* **2013**, *5*, 2861.

[5] Lindhorst, T. K.; Kotter, S.; Krallmann-Wenzel, U.; Ehlers, S. *J. Chem. Soc., Perkin Trans., 1* **2001**, 823.

[6] Otman, O.; Boullanger, P.; Drockenmuller, E.; Hamaide, T. *Beilstein J. Org. Chem.* **2010**, *6*, 58.

[7] Susumu, K.; Mei; B. C.; Mattoussi, H. *Nat. Protoc.* **2009**, *4*, 424.

[8] Saha, S. K.; Brewer, C. F. *Carbohydrate Res.* **1994,** *254*, 157.

[9] Hill, H.D.; Millstone, J. E.; Banholzer, M. J.; Mirkin, C.A. *ACS Nano* **2009**, *3*, 418.

[10] Mitchell, D. A.; Fadden, A. J.; Drickamer K. *J. Biol. Chem.* **2001**, 276, 28939.

[11] Feinberg, H.; Guo, Y.; Mitchell, D. A.; Drickamer K.; Weis, W. I. *J. Biol. Chem.* **2005**, 280, 1327.

[12] Bruckbauer, A.; Zhou, D.; Ying, L.; Korchev, Y.E.; Abell, C.; Klenerman, D. *J. Am. Chem. Soc.* **2003**, 125, 9834.

[13] Zhang, Y.; Zhang, H.; Hollins, J.; Webb, M.E.; Zhou, D. *Phys. Chem. Chem. Phys*. **2011**, *13*, 19427.

[14] Ying, L. M.; Green, J. J.; Li, H.T.; Klenerman, D.; Balasubramanian, S. *Proc. Natl. Acad. Sci. USA*. **2003**, *100*, 14629.

[15] Dahlmann, F.; Biedenkopf, N.; Babler, A.; Jahnen-Dechent, W.; Karsten, C. B.; GnirSs, K.; Schneider, H.; Wrensch, F.; O'Callaghan, C. A.; Bertram, S.; Herrler, G.; Becker, S.; Pohlmann, S.; Hofmann-Winkler, H. *J. Infect. Dis.* **2015**, *212 Suppl 2*, S247.

[16] Charbonniere, L. J. & Hildebrandt, N. *Eur. J. Inorg. Chem.* **2008**, 3241.
